# Low-temperature synthesis of cation-ordered bulk Zn_3_WN_4_ semiconductor *via* heterovalent solid-state metathesis†

**DOI:** 10.1039/d4sc00322e

**Published:** 2024-05-15

**Authors:** Christopher L. Rom, Shaun O'Donnell, Kayla Huang, Ryan A. Klein, Morgan J. Kramer, Rebecca W. Smaha, Andriy Zakutayev

**Affiliations:** a Materials, Chemical, and Computational Science, National Renewable Energy Laboratory Golden CO 80401 USA christopher.rom@nrel.gov andriy.zakutayev@nrel.gov; b Department of Chemistry, Colorado State University Fort Collins CO 80523 USA; c University of Illinois Urbana-Champaign Champaign IL 61801 USA; d Center for Neutron Research, National Institute of Standards and Technology Gaithersburg MD 20899 USA; e Department of Chemistry, Southern Methodist University Dallas TX 75275 USA

## Abstract

Metathesis reactions are widely used in synthetic chemistry. While state-of-the-art organic metathesis involves highly controlled processes where specific bonds are broken and formed, inorganic metathesis reactions are often extremely exothermic and, consequently, poorly controlled. Ternary nitrides offer a technologically relevant platform for expanding synthetic control of inorganic metathesis reactions. Here, we show that energy-controlled metathesis reactions involving a heterovalent exchange are possible in inorganic nitrides. We synthesized Zn_3_WN_4_ by swapping Zn^2+^ and Li^+^ between Li_6_WN_4_ and ZnX_2_ (X = Br, Cl, F) precursors. The *in situ* synchrotron powder X-ray diffraction and differential scanning calorimetry show that the reaction onset is correlated with the ZnX_2_ melting point and that product purity is inversely correlated with the reaction's exothermicity. Therefore, careful choice of the halide counterion (*i.e.*, ZnBr_2_) allows the synthesis to proceed in a swift but controlled manner at a surprisingly low temperature for an inorganic nitride (300 °C). High resolution synchrotron powder X-ray diffraction and diffuse reflectance spectroscopy confirm the synthesis of a cation-ordered Zn_3_WN_4_ semiconducting material. We hypothesize that this synthesis strategy is generalizable because many Li–M–N phases are known (where M is a metal) and could therefore serve as precursors for metathesis reactions targeting new ternary nitrides. This work expands the synthetic control of inorganic metathesis reactions in a way that will accelerate the discovery of novel functional ternary nitrides and other currently inaccessible materials.

## Abstract 2

Metathesis reactions can synthesize a semiconductor Zn_3_WN_4_ from Li_6_WN_4_ combined with a ZnX_2_ salt (where X = Br, Cl, F).

## Graphical abstract

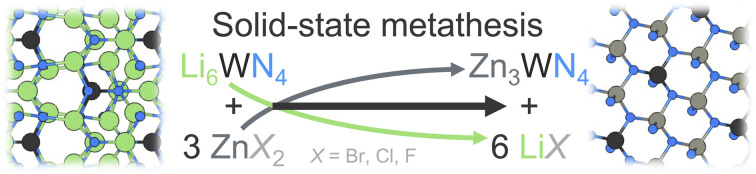

## Introduction

Metathesis is a powerful synthetic tool used across a wide range of chemistries. In metathesis reactions, a desired product BQ is synthesized following the general form, AQ + BX → BQ + AX, where the units A and B trade partners and AX is an (ideally separable) byproduct. Initial reports of metathesis reactions date back to 1925, where binary sulfides were synthesized *via* inorganic solid-state reactions: *e.g.*, ZnS + CdO → CdS + ZnO.^1^ Subsequently, catalyzed olefin metathesis was discovered in the 1950's, and decades of work led to deep mechanistic understanding, expansive synthetic control, and ultimately, to the 2005 Nobel Prize in Chemistry.^2–6^ Presently, the technique enables synthetic precision for organic and organometallic chemistry,^7–11^ metal–organic-frameworks,^12–14^ polymers,^15–18^ and beyond. In contrast, research on inorganic solid-state metathesis mostly focused on rapid, highly exothermic syntheses for binary compounds in the 1990's and early 2000's.^19^ Despite the historic head-start for inorganic solid-state metathesis, synthetic control is nascent.^20,21^

Ternary nitrides provide a prime set of materials for expanding the synthetic control of metathesis reactions. Ternary nitrides are a promising class of semiconductors,^22^ yet relatively few are known. This dearth of nitrides is primarily due to the synthetic challenges of realizing these materials from elemental metal (or binary) precursors and dinitrogen gas.^22–25^ Molecular (di)nitrogen, N_2_, is highly stable, and high temperatures are needed to break the strong N≡N triple bond (945 kJ mol^−1^).^26^ High temperatures (>800 °C) are also needed to drive diffusion, as nitrides tend to have high cohesive energies (*i.e.*, strong M–N bonds) and slow diffusion.^27–29^ Moreover, entropic penalties disfavor nitride incorporation in solids at high temperatures (*i.e.*, gaseous N_2_ is favored). Finding a synthesis temperature that is hot enough for reactivity but cool enough to avoid decomposition is therefore challenging. Adding to the difficulty, O_2_ is more reactive towards most metals than N_2_, so syntheses must be conducted in rigorously air-free conditions to avoid the formation of oxide impurities. Consequently, the number of known ternary nitrides lags behind the ternary oxides by an order of magnitude.^22–25^ Developing new synthesis methods (*e.g.*, controlled metathesis reactions) will help narrow this disparity, and in doing so, discover new materials upon which improved technologies can be built.

Zn-containing ternary nitrides epitomize the promising applications and synthetic challenges of this class of materials. Fully nitridized compounds like ZnSnN_2_ and Zn_3_WN_4_ (with metals in the highest oxidation state) are of interest as semiconductors for their high earth abundance and tunable bandgaps (spanning *ca.* 1 eV for ZnSnN_2_ to 4 eV for Zn_3_WN_4_).^30,31^ However, the bulk synthesis techniques that have been reported for Zn–M–N phases are limited to traditional ceramic methods (*i.e.*, metals + N_2_ or NH_3_ at high temperatures) or high-pressure solid state metathesis reactions (*e.g.*, 2 Li_3_N + ZnF_2_ + SnF_4_ → ZnSnN_2_ + 6 LiF).^32,33^ These bulk methods have only produced fully nitridized phases when M is a main group element (*i.e.*, LiZnN, Ca_2_ZnN_2_, Sr_2_ZnN_2_, Ba_2_ZnN_2_, ZnSiN_2_, ZnGeN_2_, ZnSnN_2_).^28,30–40^ When transition metals are used in bulk syntheses, they tend to form sub-nitrides: *e.g.*, Ti_3_ZnN_0.5_, V_3_Zn_2_N, Ti_2_ZnN, Mn_3_ZnN, and Fe_3_ZnN.^41–46^ The nitrogen-poor nature of these materials stems from the challenges described above (*i.e.*, N_2_ stability, slow diffusion). Synthesizing fully-nitridized Zn–M–N (where M is a transition metal) in bulk would advance technologies in which thin film nitrides have already shown promise, like photoelectrochemical energy conversion (ZnTiN_2_),^47^ transparent conducting oxides (ZnZrN_2_),^48^ and non-linear optics (Zn_3_WN_4_).^49^

Synthesizing Zn–M–N ternary nitrides *via* traditional methods is difficult. Many transition metals are highly refractory, meaning high temperatures would likely be needed for interdiffusion of reactants. However, Zn has a low melting point (419 °C) and a relatively low boiling point (907 °C),^50^ meaning that high temperatures would volatilize Zn away from the other metal unless special measures were taken (*e.g.*, high pressure, closed vessels). Forming binary nitrides to use as precursors instead of metals is also challenging: Zn (like other late-transition metals) does not react with N_2_ at elevated temperatures, so Zn_3_N_2_ must be synthesized under ammonia.^51^ And as noted in thin film work, fully nitridized transition metal Zn–M–N phases have low decomposition temperatures on the order of 600–700 °C.^24,47,48,52^ These challenges mean that bulk synthesis of Zn–M–N from the elements or binaries would likely proceed only at low temperatures and extremely slowly, unless special high-pressure methods were employed (*e.g.*, ammonothermal synthesis,^33^ diamond anvil cell synthesis,^53^*etc.*).

Metathesis reactions are one promising way to circumvent the challenge of diffusion in the solid state.^54^ To synthesize nitrides, this strategy starts with one nitrogen-containing precursor and one halide precursor, rather than elements or binary nitrides. The balanced reaction targets the desired phase along with a byproduct (often a halide salt). The formation of this byproduct provides a large thermodynamic driving force for the reaction and (ideally) can be washed away post-reaction. For example, Kaner *et al.* showed that mixing Li_3_N with metal chlorides would produce LiCl in highly exothermic metathesis reactions that yielded a range of binary nitrides^55–63^ and some ternary nitrides.^64,65^ Alternatively, less exothermic reactions can be conducted with greater synthetic control,^66–70^ including low-temperature topotactic reactions (*T*_rxn_*ca.* 200–400 °C).^71–73^ As for Zn–M–N compounds, ZnSnN_2_ and ZnSiN_2_ have been made using high pressure metathesis reactions, where the pressure is necessary to the loss of avoid gaseous N_2_.^32,40^ Metathesis is well known for “turning down the heat” in solid-state synthesis^74^ but is underutilized for synthesizing nitrides.

Here, we synthesize Zn_3_WN_4_*via* a near-topotactic metathesis reaction between Li_6_WN_4_ and ZnX_2_ (X = Br, Cl, F) at 300 °C and low pressure. *In situ* synchrotron powder X-ray diffraction (SPXRD) paired with differential scanning calorimetry (DSC) measurements reveal the reaction pathways and show that using a ZnBr_2_ precursor is preferable over the fluoride or chloride analogs. High resolution SPXRD measurements indicate that the Zn_3_WN_4_ product is a mostly cation-ordered structure in space group *Pmn*2_1_. We report some preliminary property characterizations for Zn_3_WN_4_, revealing optical absorption onsets near 2.5 eV and 4.0 eV, as well as paramagnetism consistent with some degree of disorder and off-stoichiometry. The reaction is near-topotactic, in that the structures of the Li_6_WN_4_ precursor and the Zn_3_WN_4_ product are related by a shift in anion layers but the [WN_4_] tetrahedral unit is preserved. Using this synthesis approach, we also synthesized Zn_3_MoN_4_, albeit with lower levels of purity in our un-optimized reactions. This work demonstrates the viability of Li–M–N phases as metathesis precursors to synthesize other ternary nitride compounds, expanding the toolkit for materials discovery.

## Results and discussion

### *In situ* SPXRD measurements

Zn_3_WN_4_ was successfully synthesized *via* metathesis reactions. The net reaction is:Li_6_WN_4_ + 3 ZnX_2_ → Zn_3_WN_4_ + 6 LiX (X = Br, Cl, F)

**Fig. 1:**
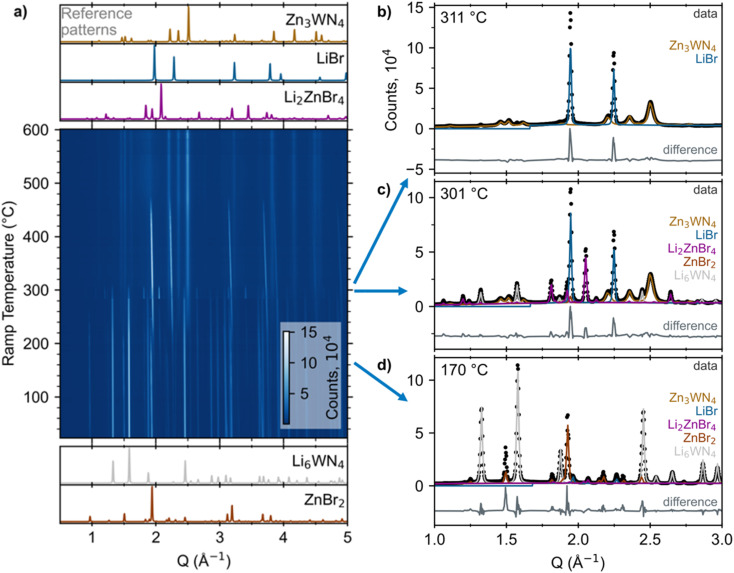
(a) Heatmap of *in situ* SPXRD data upon heating a sealed capillary of 3 ZnBr_2_ + Li_6_WN_4_ at +10 °C min^−1^. Reference patterns for the reactants and products/intermediates are simulated at the bottom and top, respectively (ICSD Col. Codes 30803 for ZnBr_2_, 66096 for Li_6_WN_4_, 73223 for Li_2_ZnBr_4_, and 53819 for LiBr).^75–77^ Analogous plots of the ZnCl_2_ and ZnF_2_ reactions are in Fig. S2 and S3.† Select patterns and fits are shown for ramp temperatures of (b) 311 °C, (c) 301 °C, and (d) 170 °C. Contributions from each phase (determined *via* Rietveld fitting) are displayed as colored lines. Difference traces are offset for clarity.

*In situ* SPXRD measurements reveal that Li_6_WN_4_ (synthesized by a ceramic method, Fig. S1†) directly converts to Zn_3_WN_4_*via* metathesis without intermediate nitride phases or solid solution behavior as a function of temperature (Fig. 1, S2 and S3†). However, the halide precursor exerts an influence on the reaction kinetics and thermodynamics, which ultimately impact the reaction pathway and final product purity.

**Fig. 2:**
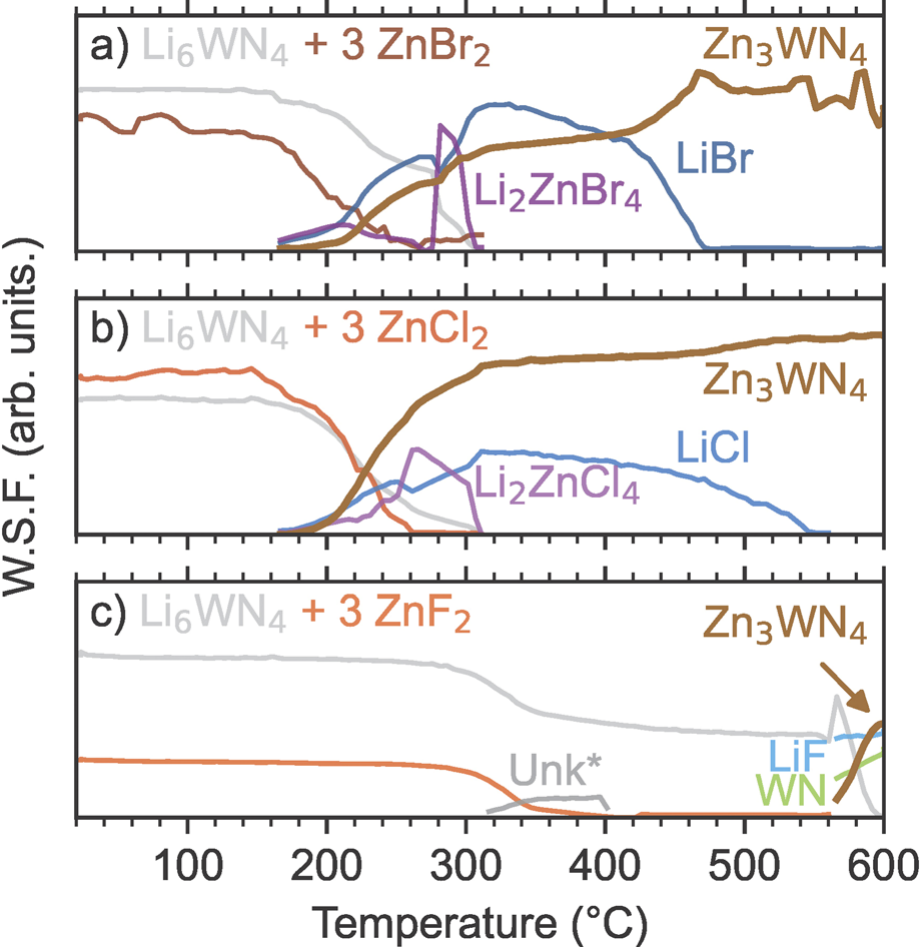
Sequential Rietveld analysis of *in situ* SPXRD patterns yields the weighted scale factors (W.S.F.), which are plotted as a function of temperature. The W.S.F. indicates the relative phase concentrations for the crystalline phases during reactions between Li_6_WN_4_ and (a) ZnBr_2_, (b) ZnCl_2_, and (c) ZnF_2_. One unknown phase (Unk*, likely a Li–Zn–F phase) is modeled using a peak fit to the most intense reflection, rather than full-pattern Rietveld analysis.

*In situ* SPXRD measurements reveal that the reaction pathway proceeds without intermediate nitrides between Li_6_WN_4_ and Zn_3_WN_4_. Fig. 1a shows a heatmap for X = Br as an example; subsequent examination revealed that this anion leads to the most phase pure product. The reaction of Li_6_WN_4_ + 3 ZnBr_2_ → Zn_3_WN_4_ + 6 LiBr initiates near 170 °C and proceeds to completion within the 14 minutes of ramp time up to 310 °C (Fig. 1b). Near 170 °C, the Bragg peaks arising from crystalline Li_6_WN_4_ and ZnBr_2_ begin to gradually decline in intensity. Shortly thereafter, new sets of Bragg peaks that can be indexed to LiBr, Li_2_ZnBr_4_, and Zn_3_WN_4_ begin growing in intensity (Fig. 1d). The Bragg peaks corresponding to Li_2_ZnBr_4_ gradually decrease in intensity between 210 °C and 270 °C, increase dramatically in intensity at 275 °C, and then disappear entirely at 305 °C (Fig. 1c). Such fluctuations may stem from crystal nucleation and growth within the capillary, especially given the small spot size of the synchrotron X-ray beam, possibly combined with crystallite motion in a liquid-like medium. Diffraction images show spotty diffraction patterns, consistent with crystallite growth. These data indicate that the synthesis proceeds directly *via* Li_6_WN_4_ + 3 ZnBr_2_ → Zn_3_WN_4_ + 6 LiBr. While this process occurs, the metal halides also react with one another: 2 LiBr + ZnBr_2_ → Li_2_ZnBr_4_. We do not observe signs of a crystalline theoretically-predicted LiZn_4_W_2_N_7_ structure,^78^ although this does not rule out the presence or synthesizability of such a phase. Similar trends are noted with the ZnCl_2_ and ZnF_2_ reactions (Fig. S2 and S3†), as shown by sequential Rietveld analysis (Fig. 2).

Sequential Rietveld analysis of *in situ* variable temperature SPXRD measurements of the Li_6_WN_4_ + 3 ZnX_2_ reactions shows that the ZnBr_2_ and ZnCl_2_ reactions initiate at much lower temperatures than the ZnF_2_ reaction (Fig. 2). For both the ZnBr_2_ and ZnCl_2_ reactions (Fig. 2a and b), the concentrations of the precursor phases start decreasing near 170 °C, followed shortly thereafter by Zn_3_WN_4_ and LiX formation and growth. At the same time, ternary halides Li_2_ZnBr_4_ and Li_2_ZnCl_4_ form, and are then consumed or melt near 300 °C. In contrast, the precursors of the ZnF_2_-based reaction do not begin declining until over 300 °C (Fig. 2c). The concomitant decrease in Li_6_WN_4_ and ZnF_2_ suggests reactivity, but neither Zn_3_WN_4_ nor LiF are detected in our data at this temperature. Instead, very weak reflections for an unknown phase appear in the data (labeled as Unk*). This phase may be a Li–Zn–F ternary, but it does not index to any known ternary fluoride unit cells, including the reported Li_2_ZnF_4_ phase.^79^ An amorphous phase is likely present in the 400 to 570 °C region, given the decrease in precursor peaks and lack of new intermediate peaks. Zn_3_WN_4_ and LiF crystallize above 570 °C, along with a rocksalt phase (fit with WN, but the material may be a (Zn,W)N_*x*_ phase as observed with the Mo-based system, Fig. S4†). We did not study the ZnF_2_ reactions further, given that phase-pure Zn_3_WN_4_ did not crystallize and given the challenge associated with washing away LiF from the product. Instead, we focus on the ZnBr_2_ and ZnCl_2_ reactions.

### DSC measurements

**Fig. 3:**
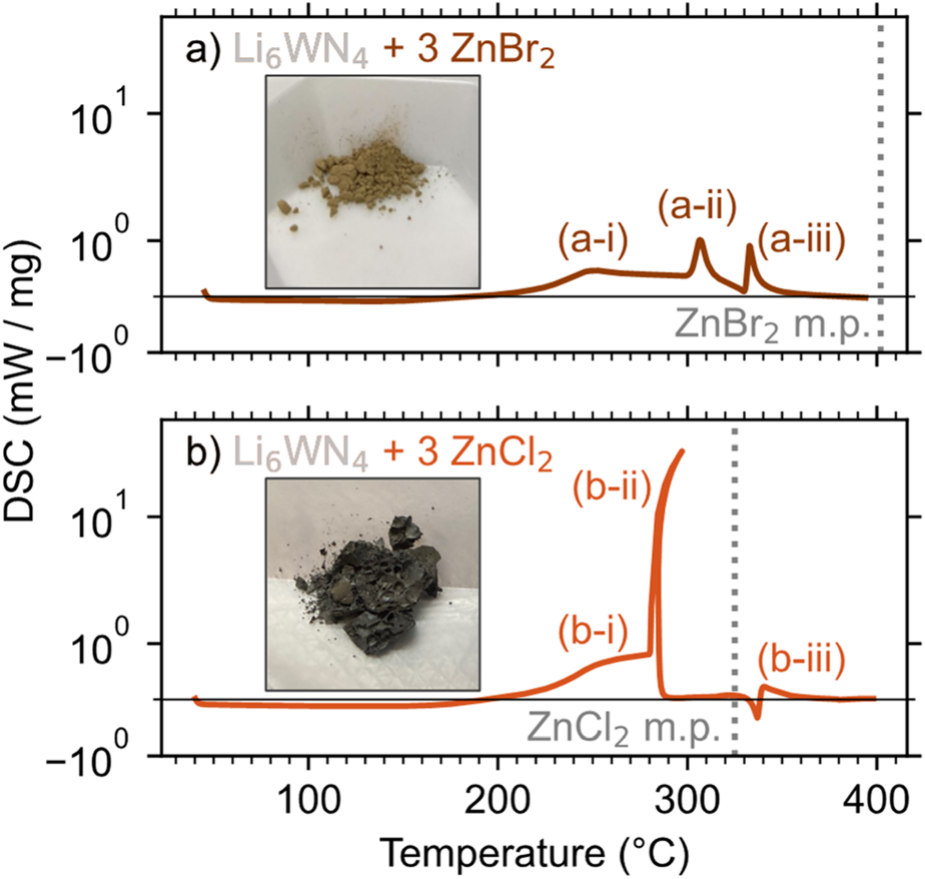
DSC measurements of Li_6_WN_4_ reacting with (a) 3 ZnBr_2_ and (b) 3 ZnCl_2_. Inset photos show the products from reactions heated at 300 °C for 1 h.

DSC was employed to study the ZnBr_2_- and ZnCl_2_-based reactions, with reaction mixtures sealed in aluminum pans under argon for the measurements. The lower exothermicity of the ZnBr_2_-based reaction leads to a more controlled release of heat and greater product purity, compared to the ZnCl_2_-based reaction. DSC measurements show that the ZnBr_2_ reaction has three small exotherms (Fig. 3a). A gradual exotherm starts near 190 °C (a-i), followed by two exotherms near 305 °C (a-ii) and 334 °C (a-iii). Peak a-i is likely a slow, solid–solid reaction of Li_6_WN_4_ + 3 ZnBr_2_ → Zn_3_WN_4_ + 6 LiBr, consistent with the *in situ* SPXRD measurement. Peak a-ii may be the reaction 2 LiBr + ZrBr_2_ → Li_2_ZnBr_4_. Li_2_ZnBr_4_ melts at 326 °C,^77^ so peak a-iii is likely a rapid completion of the Zn_3_WN_4_ formation facilitated by the presence of a liquid phase. This kind of rapid exothermic event is common in metathesis reactions; once a liquid phase forms, reaction kinetics accelerate and the heat release self-propagates.^57^

The ZnCl_2_ reaction (Fig. 3b) starts similarly, with a gradual exotherm between 190 °C and 280 °C (b-i), a solid–solid reaction yielding Zn_3_WN_4_ and LiCl. Then at ∼280 °C, a massive exotherm (b-ii) initiates just below the melting point of ZnCl_2_ (325 °C). This event likely corresponds to the formation of a liquid phase, such as a LiCl–ZnCl_2_ eutectic (275 °C at 78% ZnCl_2_ and 287 °C at 91% ZnCl_2_).^80^ Peak b-ii has curvature because this event releases heat so quickly that the DSC stage increases in temperature by approximately 15 °C, after which the DSC pan cools slightly (Fig. S5†). Lastly, a small endotherm is observed at 336 °C (b-iii), consistent with the melting of Li_2_ZnCl_4_. These results are broadly consistent with the *in situ* SPXRD results, albeit shifted slightly in temperature owing to differences in experimental configuration.

These DSC results show why the ZnBr_2_ reaction yields the purest product while the ZnCl_2_ reaction exhibits a small Zn impurity. The rapid release of heat in the ZnCl_2_ reaction causes small portions of the material to decompose: Zn_3_WN_4_ → W + 3 Zn + 2 N_2_ or Zn_3_WN_4_ → WN + 3 Zn + 3/2 N_2_ (Fig. S6†). In contrast, the washed product of the ZnBr_2_ synthesis yielded a PXRD pattern with all Bragg peaks indexed to *Pmn*2_1_ Zn_3_WN_4_. These differences can easily be seen in the color of the material (see insets, Fig. 3), where Zn impurities in the ZnCl_2_ reaction led to a grey color. The Zn_3_WN_4_ sample produced by the ZnBr_2_ reaction is light brown and was phase pure (as discussed subsequently). These differences stem from both thermodynamic and kinetic factors.

### Thermodynamic and kinetic factors

**Fig. 4:**
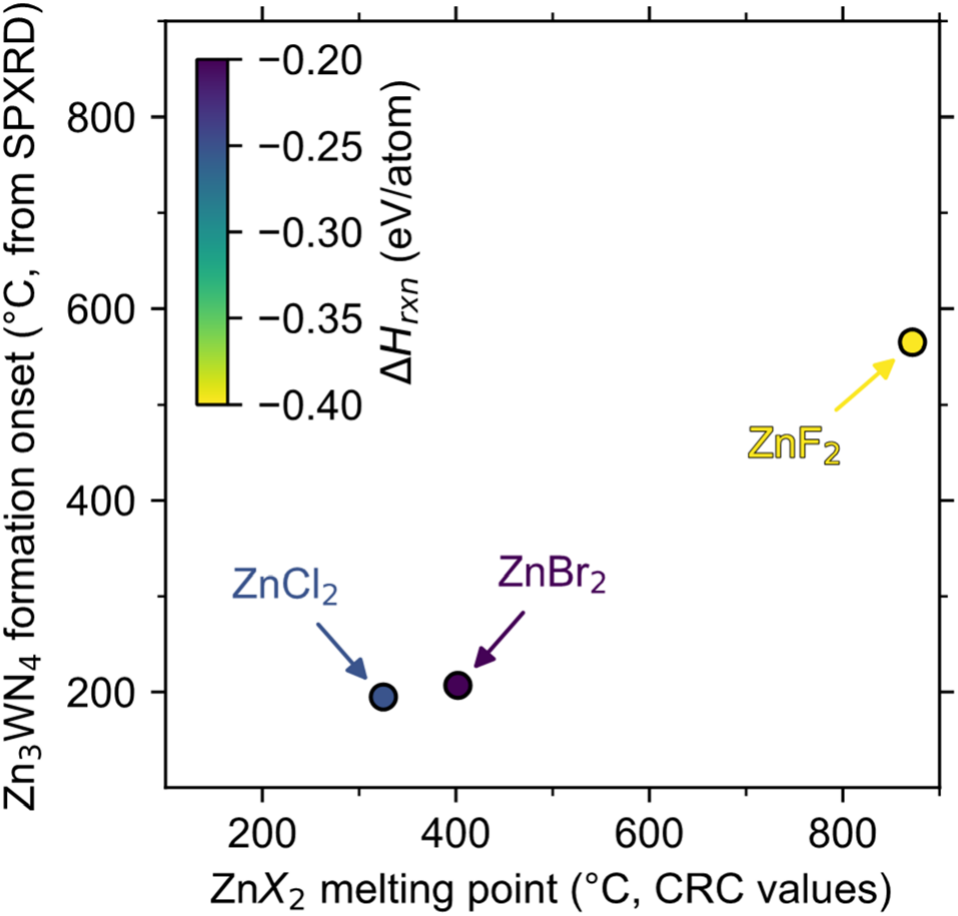
The initial formation of Zn_3_WN_4_ (as detected by *in situ* SPXRD) is correlated with the melting point of the halide salt (from CRC values)^50^ but is not correlated with calculated reaction enthalpy (Δ*H*_rxn_, color, from the materials project).^84^ The dashed line shows where melting point would equal onset temperature.

Zn_3_WN_4_ is calculated to be thermodynamically stable at moderate temperatures, so differences in product purity reflect differences in kinetic control for the ZnX_2_ reactions. For each halide, the reactions are calculated to be enthalpically driven (*i.e.*, negative Δ*H*_rxn_, Fig. 4). Furthermore, chemical potential diagrams show that Li_6_WN_4_ and Zn_3_WN_4_ share a border in chemical potential space (Fig. S7†), indicating that diffusion can occur between these phases without nucleating an intermediate phase at the interface,^81^ as previously shown for metathesis reactions towards ternary oxides and nitrides.^70,82^ However, Zn_3_WN_4_ is only stable down to a nitrogen chemical potential of *μ*_N_ = −0.26 eV when at 300 °C (Fig. S7a and b†), whereas these reactions were conducted in evacuated ampules (*p* < 0.03 torr, *μ*_N_ < −0.5 eV). This means thermodynamics favor some N_2_ (g) evolution from the reaction (with Zn and W as decomposition products). Therefore, the fact that the ZnBr_2_ reaction proceeds without detectable decomposition means the reaction is fully under kinetic control, while the ZnCl_2_ and ZnF_2_ reactions are not.

The magnitude of the exothermicity combined with the melting points of the halide salts affect the degree of kinetic control. The lowest melting point we can identify in the LiBr–ZnBr_2_ system is 326 °C for Li_2_ZnBr_4_,^77^ meaning the reaction may be controlled by solid-state diffusion below this point. Additionally, the Δ*H*_rxn_ of the ZnBr_2_ reaction (−0.20 eV per atom) is less exothermic than the ZnCl_2_ reaction (−0.25 eV per atom), which may limit local self-heating and avoid decomposition (Fig. 3a). In contrast, the liquidus line in the LiCl–ZnCl_2_ system extends as low as 275 °C,^80^ at which point diffusion accelerates rapidly (Fig. 3b, peak b-ii). Even if the temperature is kept lower (*e.g.*, 250 °C), partial Zn_3_WN_4_ decomposition is observed in the ZnCl_2_ system (Fig. S6†). This decomposition indicates a loss of kinetic control. The ZnF_2_ reaction is worse. The high melting point of ZnF_2_ (872 °C)^50^ means the kinetics are sluggish until the reaction is perilously close to the decomposition temperature (estimated near 700 °C, Fig. S7c and d†), at which point the self-heating from the high exothermicity (Δ*H*_rxn_ = −0.40 eV per atom)^83^ likely drives substantial decomposition (Fig. 2c). In contrast, the ZnBr_2_-based reaction retains kinetic control as the moderate melting point of Li–Zn–Br phases enable diffusion near 300 °C while the low Δ*H*_rxn_ prevents excessive self-heating, thereby avoiding decomposition.

This type of reaction control has been explored in oxides but is less well studied for ternary nitrides. “Spectator ions” that are not incorporated into the final product still have substantial influence over reaction pathways and polymorph formation, as demonstrated for syntheses of Y–Mn–O phases.^21,82,85–88^ In particular, work on “co-metathesis” identified that when eutectic halide mixtures form *in situ*, these liquids decrease reaction onset temperatures relative to systems without eutectics.^85,86^ Similar eutectics are likely forming between ZnX_2_ and LiX in our syntheses of Zn_3_WN_4_. This thermodynamic analysis, along with *in situ* SPXRD and DSC measurements, guided our optimization of the synthesis for Zn_3_WN_4_.

### Structural and composition analysis of Zn_3_WN_4_

The best conditions we found for the synthesis of Zn_3_WN_4_ were heating ZnBr_2_ with Li_6_WN_4_ at a ramp of +5 °C min^−1^ to 300 °C for a 1 h dwell, followed by natural cooling in the furnace. This reaction was scaled up to *ca.* 1 g of reactant mix for *ex situ* analysis. Washing with anhydrous methanol successfully removed byproduct LiBr and excess ZnBr_2_ while preserving the targeted phase. We subsequently learned that Zn_3_WN_4_ does not appear to be moisture or air sensitive, so washing with water may be viable in future work. We used a slight excess of ZnBr_2_ (3.1 ZnBr_2_ + Li_6_WN_4_) to ensure complete conversion and minimize reaction temperature by acting as a heat sink. XRF measurements show a Zn : W ratio of 3.165(3) : 1, slightly higher than the expected 3 : 1 ratio of Zn_3_WN_4_ (representative XRF spectrum shown in Fig. S8†), which may be a result of the excess ZnBr_2_. PXRD techniques confirmed that this synthesis of Zn_3_WN_4_ proceeded without the formation of decomposition products (*i.e.*, Zn, (Zn,W)N_*x*_ phases).

**Fig. 5:**
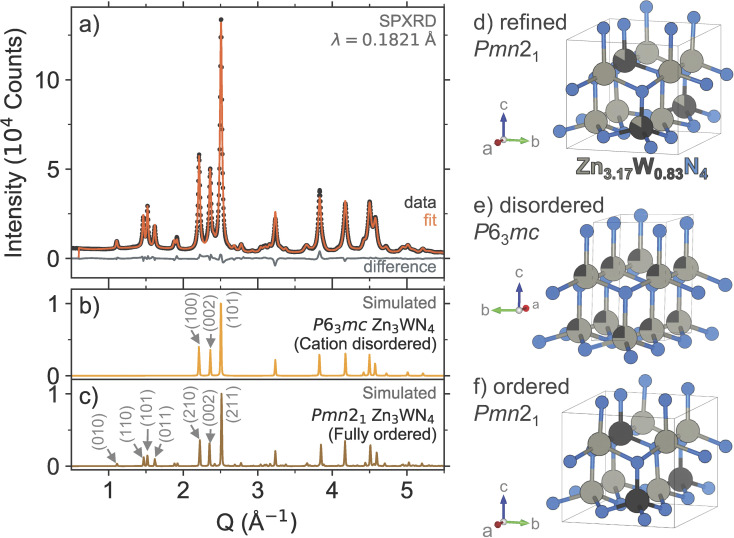
(a) High resolution SPXRD data (black dots) and Rietveld refinement (orange trace) of Zn_3_WN_4_ powder. Simulated patterns are shown for reference: (b) the cation-disordered *P*6_3_*mc* model and (c) the fully ordered *Pmn*2_1_ model. Visualizations of (d) the *Pmn*2_1_ structure of Zn_3_WN_4_ refined from the SPXRD data, (e) the cation-disordered *P*6_3_*mc* model, and (f) the fully ordered *Pmn*2_1_ model.

High resolution SPXRD measurements confirm the successful synthesis of Zn_3_WN_4_ (Fig. 5). Rietveld analysis of the SPXRD data (Fig. 5a) shows that Zn_3_WN_4_ crystallizes in space group *Pmn*2_1_ with lattice parameters *a* = 6.5602(8) Å, *b* = 5.6813(7) Å, and *c* = 5.3235(2) Å. The presence of intensity at the (010), (110), (101), and (011) Bragg positions indicates a substantial degree of cation ordering (Fig. 5c and f). The peaks for the (210), (002), and (211) reflections are characteristic of wurtzite-derived structures; these correspond to the (100), (002), and (101) reflections in the prototypical wurtzite structure (*P*6_3_*mc*), respectively (Fig. 5b and e). Rietveld-refined occupancies suggest a Zn : W ratio of 3.8 : 1, a higher ratio than that measured by XRF (3.2 : 1), with partial occupancy of Zn on the W site (Table S1†), indicating a composition of Zn_3.17_W_0.83_N_4_ (Fig. 5d). The occupancies of the N atoms refined to 1 within error and so were fixed at unity. Alternative structural models were also considered, as discussed further in the ESI (Table S2 and Fig. S9–S11).† We selected the single-phase model shown in Fig. 5 as it is the simplest model that effectively describes both the diffraction data (presented here) and the optical data (discussed subsequently).

Our metathesis approach yielded a different polytype for Zn_3_WN_4_ compared to prior thin film syntheses. Metathesis between Li_6_WN_4_ and ZnBr_2_ successfully synthesized Zn_3_WN_4_ in space group *Pmn*2_1_ (Fig. 5). In contrast, prior thin film sputtering work produced cation-disordered *P*6_3_*mc* structures.^23,52,89^ While both the *Pmn*2_1_ and *P*6_3_*mc* structures are wurtzite-derived, the cation-ordered structure is expected to be the thermodynamic ground state.^23,48^ In thin film sputtering, high-energy plasma precursors deposit onto a substrate and quench rapidly in a local energy minimum, thus locking in the disordered cation arrangement.^48^ While bulk syntheses can sometimes lead to cation-disordered structures,^28,32,66,67,70^ the high charge on W(6+) likely encourages ordering to maximize the spacing between the hexavalent cations. The reaction pathway here proceeds in a way that avoids the local energy minimum of the disordered structure, instead forming a (mostly) ordered structure. This aspect of the bulk synthesis may be due to the ordered nature of W^6+^ in the Li_6_WN_4_ precursor. Further work is needed to assess the influence of reaction conditions (*e.g.*, precursor ratios, halide choice, heating profiles, *etc.*) on cation ordering, as the degree of cation ordering affects the optical properties of the material.

### Property measurements

**Fig. 6:**
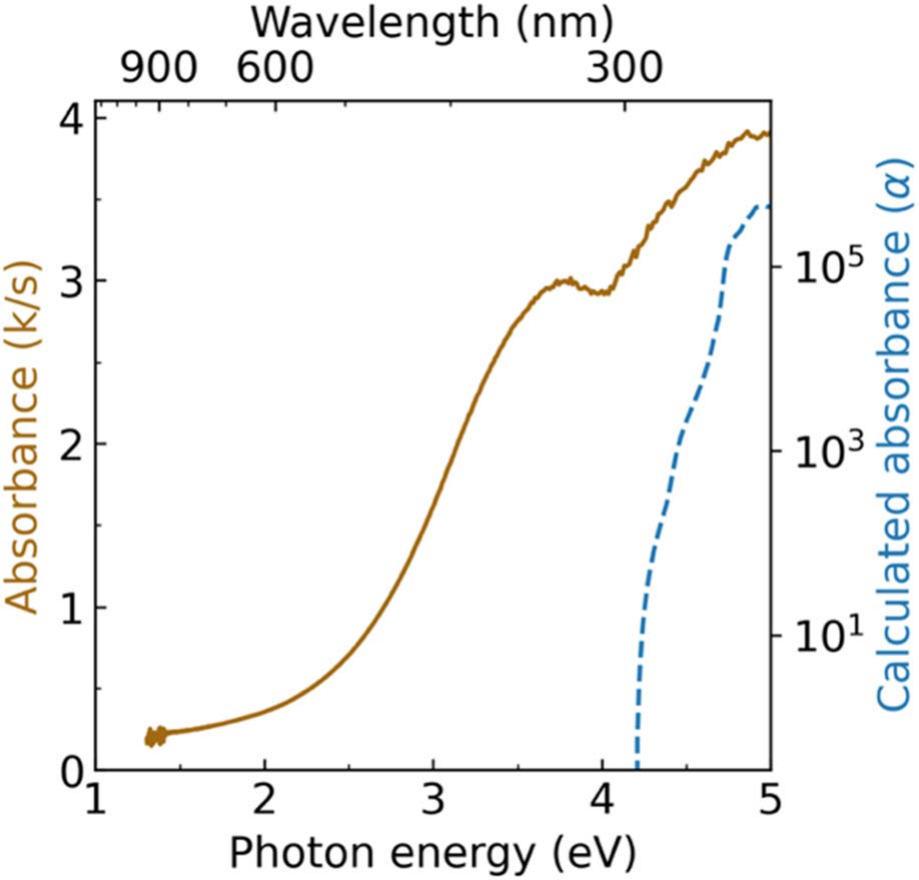
Diffuse reflectance spectrum of Zn_3_WN_4_ (solid brown trace) compared with GW-calculated absorbance (dashed blue trace).

Diffuse reflectance spectroscopy measurements reveal two absorption onsets for Zn_3_WN_4_: one near 2.5 eV and another near 4.0 eV (Fig. 6). The absorption feature near 4.0 eV is consistent with the expected bandgap for long-range cation-ordered Zn_3_WN_4_ (*i.e.*, the *Pmn*2_1_ space group). The GW-calculated indirect band gap for this phase is 3.96 eV, with a direct bandgap of 4.20 eV (NREL MatDB ID 287103; blue trace in Fig. 6).^90,91^ Other researchers using a hybrid functional, HSE06, calculated the bandgap to be 3.60 eV.^49^ The absorption feature near 2.5 eV is similar to the 2.0 eV to 2.4 eV absorption onset reported for cation disordered Zn_3_MoN_4_ and Zn_3_WN_4_ synthesized as thin films.^52,89^ Computational work on similar materials has shown that localized, mid-gap electronic states arise when N atoms have Zn-rich coordination.^92^ These two optical features are consistent with the Rietveld analysis of our Zn_3_WN_4_ powder sample (Fig. 5): long-range cation order is present (leading to the 4.0 eV absorption) along with some Zn anti-site defects on the W position (which lead to Zn-rich coordination for N and the 2.5 eV absorption). Magnetic susceptibility measurements are consistent with the presence of a paramagnetic impurity, as the material does not exhibit purely diamagnetic behavior (Fig. S12†). We note that the same batch of Zn_3_WN_4_ was used for the diffuse reflectance optical spectroscopy, magnetometry, XRF, and the high resolution SPXRD measurements.

### Structural relation of precursor and product

**Fig. 7:**
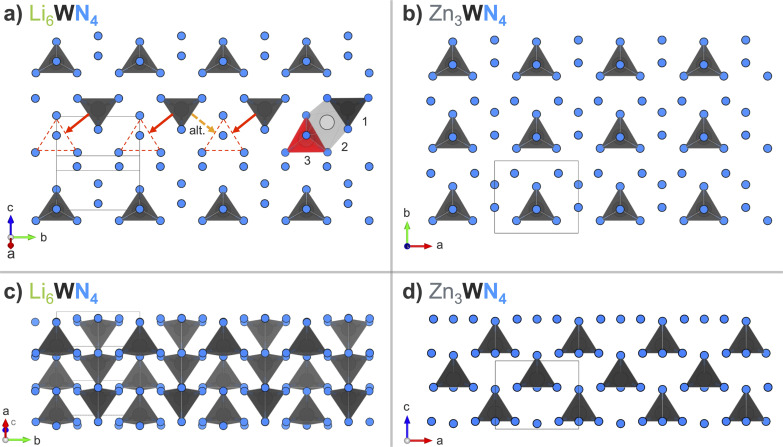
Arrangement of [WN_4_] tetrahedral units when looking down (a) on one layer of Li_6_WN_4_ (*P*4_2_/*nmc*) in the (201) plane, and (b) on a layer of Zn_3_WN_4_ (*Pmn*2_1_) in the (001) plane. Red annotations in (a) show the likely displacement undergone by some W^6+^ ions in the transition to Zn_3_WN_4_*via* a tetrahedral (1) to octahedral (2) to tetrahedral (3) sequence, while the orange dashed arrow shows an alternative migration direction. Side views of three layers of (c) Li_6_WN_4_ and (d) Zn_3_WN_4_ stacked along the [201] and [001] directions, respectively. The shading of the [WN_4_] units in (c) indicates depth. Li and Zn are omitted for clarity; they are shown in Fig. S13.†

The transformation from Li_6_WN_4_ to Zn_3_WN_4_ involves slight structural rearrangements (Fig. 7). The W^6+^ retains its tetrahedral coordination and (for the most part) its oxidation state through the process, but the orientations of the polyhedra change. The fcc anion lattice of Li_6_WN_4_ converts to the hcp anion lattice of Zn_3_WN_4_. Visual inspection of the structures using VESTA software^93^ shows that one likely path for this transformation is for half of the W^6+^ ions in Li_6_WN_4_ to migrate through an octahedral site to a new tetrahedral site in Zn_3_WN_4_ (red annotations). However, various migration pathways may be occurring during the synthesis (*e.g.*, W-migration between anion layers). Zn_3_WN_4_ has broader peaks in SPXRD data than Li_6_WN_4_ (Fig. 1 and 5), indicating shorter crystalline domain lengths. That difference may arise from the W in different sections of the Li_6_WN_4_ crystallites migrating in alternative directions (Fig. 7a, dashed orange arrow). The anion packing layers also decrease in spacing from 2.767(1) Å in Li_6_WN_4_ to 2.662(1) Å in Zn_3_WN_4_. The shortest W–W distance decreases from 4.927(1) Å to 4.646(1) Å. Lastly, the centrosymmetric structure of Li_6_WN_4_ (*P*4_2_/*nmc*) converts to a polar structure of Zn_3_WN_4_ (*Pmn*2_1_). We did not observe any signs of solid solution behavior (*i.e.*, Li_6−*x*_Zn_*x*/2_WN_4_) in the *in situ* SPXRD studies. However, solid solution behavior may be present but undetected by the *in situ* SPXRD data if it occurs on short timescales (<30 s) or small length scales (*ca.* 10 nm).

The synthesis reported here is distinct from literature on prior metathesis syntheses of nitrides in that the ions undergoing exchange have different formal charges. All prior reports on nitride metathesis reactions have exchanged ions of the same charge (*e.g.*, displacing Na^+^ with Cu^+^ in ATaN_2_, or Ca^2+^ with Mg^2+^ in A_2_Si_5_N_8_; where A represents the exchangeable cations).^68,71,72^ Here, we replace a monovalent cation (Li^+^) with a divalent cation (Zn^2+^). While such exchange has been conducted in oxides^86,87,94,95^ and sulfides (*e.g.*, 2 NaCrS_2_ + MgCl_2_ → MgCr_2_S_4_ + 2 NaCl),^96^ to the best of our knowledge this is the first report of such an exchange in nitrides. The resulting decrease in the cation : anion ratio (from 7 : 4 to 4 : 4) means that a truly topotactic replacement is unlikely to occur. However, the transformation appears to be near-topotactic.

### Generalizability to other materials

There are numerous Zn–M–N phases that have been demonstrated to be synthesizable *via* thin film sputtering but that have not yet been made in bulk. In addition to Zn_3_WN_4_,^23,89^ sputtering has been used to synthesize fully nitridized transition metal ternaries: ZnTiN_2_, ZnZrN_2_, Zn_2_VN_3_, Zn_2_NbN_3_, Zn_2_TaN_3_, and Zn_3_MoN_4_.^23,24,47,48,52^ Although computational predictions for these thin film materials find that cation-ordered structures are the thermodynamic ground state (Fig. 5f), these sputtered films tend to form in cation-disordered structure variants (Fig. 5e).^23,24^ This disorder tends to decrease the bandgap of the material by creating localized electronic states.^29,92^ Bulk syntheses could advance the development of these new semiconductors by studying the effect of structure (*e.g.*, ordering) on optoelectronic properties of these new materials.

The synthesis of Zn_3_WN_4_ from Li_6_WN_4_ and ZnX_2_ suggests a promising strategy for future materials discovery of cation-ordered heterovalent ternary nitrides *via* metathesis from lithium-based ternary nitride precursors. Lithium-based ternary nitrides are the most well-studied subset of ternary nitrides,^23^ suggesting that many Li–M–N phases exist that could be used to synthesize additional A–M–N phases *via* exchange with AX_*n*_ (where A and M are metals and X is a halide). Following our results here, X should be selected to minimize reaction energy and thus minimize the risk of decomposing the target phase *via* gaseous N_2_ loss. To demonstrate this point, we also synthesized Zn_3_MoN_4_ from Li_6_MoN_4_ and ZnBr_2_ (Fig. S4†). Zn_3_MoN_4_ was the main product, but some decomposition products were also observed, indicating that additional reaction optimization is needed. Unlike in Zn_3_WN_4,_ the ZnBr_2_-based reaction was not sufficiently low-energy to fully avoid this decomposition for Zn_3_MoN_4_. While we did not synthesize phase-pure Zn_3_MoN_4_ here, further reaction engineering, like adding NH_4_Cl to manage heat flow,^62–65^ may be able to produce phase-pure Zn_3_MoN_4_. As we found that the reaction onset temperature is correlated with AX_2_ melting point, phases with high-melting temperature precursors may be difficult to synthesize below the decomposition point of the targeted ternary. Therefore, future work should consider ways to decouple the reaction onset from the AX_2_ melting point. In sum, this work shows how Li–M–N phases can be promising precursors for accelerating the discovery of new ternary nitrides.

## Conclusions

Here, we report the bulk synthesis of cation-ordered Zn_3_WN_4_, through metathesis reactions beginning from a Li-based ternary nitride precursor: Li_6_WN_4_ + ZnX_2_ → Zn_3_WN_4_ + 6 LiX (X = Br, Cl, F). These reactions proceed directly (*i.e.*, without nitride intermediates), as measured by *in situ* synchrotron powder X-ray diffraction and differential scanning calorimetry. The reaction onset temperature correlates with the melting point of the ZnX_2_ precursor, allowing ZnCl_2_- and ZnBr_2_-based reactions to proceed at ≤300 °C. The more exothermic reactions lead to greater degrees of Zn_3_WN_4_ decomposition, meaning that the least exothermic reaction (with ZnBr_2_) is the most favorable for synthesis. The high resolution synchrotron powder X-ray diffraction data are consistent with cation-ordered Zn_3_WN_4_ (*Pmn*2_1_). This finding is distinct from prior thin film syntheses, which yielded cation-disordered *P*6_3_*mc* Zn_3_WN_4_. Diffuse reflectance spectroscopy shows that Zn_3_WN_4_ powders exhibit absorption onsets near 2.5 eV and 4.0 eV, consistent with a small degree of cation disorder in the mostly long-range ordered *Pmn*2_1_ phase. Preliminary work targeting Zn_3_MoN_4_ from Li_6_MoN_4_ and ZnBr_2_ suggests this synthesis approach may readily extend to other systems. These findings indicate that Li–M–N compounds may serve as precursors for synthesizing numerous other ternary nitrides.

## Data availability

Density functional theory calculations can be found at the NREL MatDB (https://materials.nrel.gov/). The ESI† contains additional data on experimental conditions, additional PXRD patterns, *in situ* variable temperature SPXRD measurements, DSC measurements, compositional characterization, structural models, and magnetic susceptibility measurements.

## Author contributions

Christopher L. Rom: conceptualization, investigation, formal analysis, visualization, writing – original draft preparation, writing – reviewing and editing Shaun O'Donnell: investigation Kayla Huang: investigation Ryan A. Klein: investigation, formal analysis Morgan J. Kramer: investigation, formal analysis Rebecca W. Smaha: investigation, writing – reviewing and editing Andriy Zakutayev: conceptualization, funding acquisition, supervision, writing – reviewing and editing.

## Conflicts of interest

There are no conflicts to declare.

## Supplementary Material

SC-015-D4SC00322E-s001

SC-015-D4SC00322E-s002
