# Supplementary material for: Low-temperature synthesis of cation-ordered bulk Zn3WN4 semiconductor via heterovalent solid-state metathesis
Source: Chem Sci. 2024 May 15;15(25):9709–18. doi: 10.1039/d4sc00322e (PMC11206237; doi:10.1039/d4sc00322e)
Supplement: SC-015-D4SC00322E-s001 [file SC-015-D4SC00322E-s001.pdf]

Electronic Supplementary Material (ESI) for Chemical Science.
This journal is © The Royal Society of Chemistry 2024

Supplemental Information for: Low-temperature synthesis of cation-ordered bulk Zn_3WN_4 semiconductor via heterovalent solid-state metathesis

Christopher L. Rom[1,*], Shaun O'Donnell[1,2], Kayla Huang[1,3], Ryan A. Klein[1,4], Morgan J. Kramer[4,5], Rebecca W. Smaha[1], Andriy Zakutayev[1,*]

[1] Materials, Chemical, and Computational Science, National Renewable Energy Laboratory, Golden, CO, 80401, USA
[2] Department of Chemistry, Colorado State University, Fort Collins, CO, 80523, USA
[3] University of Illinois Urbana-Champaign, Champaign, IL, 61801, USA
[4] Center for Neutron Research, National Institute of Standards and Technology, Gaithersburg, MD, 20899, USA
[5] Department of Chemistry, Southern Methodist University, Dallas, TX, 75275 USA
[*] Corresponding authors: christopher.rom@nrel.gov and andriy.zakutayev@nrel.gov

Table of Contents

Experimental **2**
- **Synthesis of Zn_3WN_4** 2
- **Synthesis of Zn_3MoN_4** 2
- ***In situ* synchrotron powder X-ray diffraction analysis** 3
- ***Ex situ* powder X-ray diffraction analysis of Zn_3WN_4** 3
- **Compositional, thermodynamic, and property measurements** 4

Additional PXRD measurements **5**
In situ variable temperature SPXRD measurements **6**
Synthesis of Zn_3MoN_4 .... **9**
DSC measurements **11**
Evidence of decomposition in the 3 $ZnCl_2$ + Li_6WN_4 reaction **12**
Compositional characterization **14**
Structural models for the high resolution SPXRD measurements **15**
Magnetic susceptibility measurements **20**
Full structure visualizations of Li_6WN_4 and Zn_3WN_4 .... **21**
References **21**

Experimental

Synthesis of Zn_3WN_4

Caution: *Metathesis reactions have the potential to rapidly release heat and gas.* N_2 *gas formation within sealed ampules can cause explosions at high temperatures, and appropriate precautions should be taken to prevent equipment damage and unsafe conditions.*

As some precursors are highly moisture sensitive, all precursors were prepared and stored in an argon-filled glovebox (O_2 < 0.1 ppm, H_2O < 0.1 ppm) unless explicitly mentioned. ZnF_2 (≥99 %, anhydrous, Alfa Aesar), $ZnCl_2$ (≥99.995%, anhydrous, Sigma Aldrich), $ZnBr_2$ (99.999%, anhydrous, Aldrich), Li_3N (≥99.5%, 80 mesh, Sigma Aldrich), W (99.95%, <1 micron powder, Thermofisher Scientific) were used as received.

Li_6WN_4 was synthesized in a method modified from that of Yuan et al.[1] Solid precursors (2.1 Li_3N + W, ca. 5 mol% excess Li_3N to account for loss by evaporation) were ground with a mortar and pestle and loaded into Zr crucibles with Zr lids (ca. 1 g loose powder). The Zr crucibles were then loaded into sacrificial quartz tubes (open on one end), which were loaded into quartz process tubes and heated in a tube furnace. Custom endcaps with quick disconnects enabled air-free transfer from the glovebox to the tube furnace (under Ar or N_2). The samples were reacted under flowing N_2 (50 sccm, 99.999% purity) with a +5 °C/min ramp followed by a 12 h dwell at 850 °C and then natural cooling after turning off the furnace. Samples were recovered into the glovebox for subsequent analysis and use. The beige-colored products were confirmed to be phase pure by powder X-ray diffraction (Figure S1).

Syntheses for Zn_3WN_4 were conducted by grinding together Li_6WN_4 with ZnX_2 (X = Cl, Br) in a ratio of approximately 1:3. The powders were pelletized with 6 mm diameter dies in an arbor press (ca. 100 mg per pellet), loaded into quartz ampules (10 mm OD, 10 mm ID, ca 10 cm length), sealed under vacuum (<0.03 Torr), and heated in a muffle furnace. The optimized synthesis for Zn_3WN_4 used a ratio of Li_6WN_4 + 3.1 $ZnBr_2$ and was scaled up to a 3 g batch, sealed in a quartz ampule under vacuum (< 0.03 Torr) and heated at +5 °C/min to 300 °C for a 1 h dwell, then allowed to cool naturally. Samples were recovered into the glovebox. Reaction byproduct LiX was washed away using anhydrous and degassed methanol that was dried over molecular sieves. For washing, centrifuge tubes were loaded with approximately 500 mg of product powder and 1.5 mL methanol. The tube was agitated with a vortex, centrifuged, and the supernatant was decanted. This wash was repeated for a total of 3 cycles. Recovered powders were dried overnight under vacuum. However, Zn_3WN_4 ultimately proved to be stable against air and water, and we note that the anhydrous washing may not be necessary.

Synthesis of Zn_3MoN_4

Just like Li_6WN_4, Li_6MoN_4 was synthesized using Li_3N and Mo (≥99.9%, 1–5 micron powder, Sigma Aldrich), following a method modified from that of Yuan et al.[1] Heating the powders at 850 °C for 12 h (with slight Li_3N excess) resulted in a phase pure Li_6MoN_4 (Figure S1b). This Li_6MoN_4 was then mixed with $ZnBr_2$, pelletized, sealed in an ampule under vacuum, and heated

at +5 °C/min to 300 °C for a 1 h dwell, followed by natural cooling. The product was then washed with anhydrous methanol.

In situ synchrotron powder X-ray diffraction analysis

In situ synchrotron powder X-ray diffraction (SPXRD) measurements were conducted at beamline 17-BM-B of the Advanced Photon Source at Argonne National Laboratory. For these experiments (λ = 0.24101 Å), the PerkinElmer plate detector was positioned 700 mm away from the sample. Homogenized precursors were packed into quartz capillaries in an Ar glovebox and flame-sealed under vacuum (<30 mTorr). Capillaries were loaded into a flow-cell apparatus[3] and heated at 5 °C/min to the specified temperature. A thermocouple was placed against the tip of the sample capillary, approximately 2 mm horizontally from the position of the X-ray beam. Diffraction pattern images were collected every 30 s by summing 20 exposures of 0.5 s each (10 s of summed exposure), followed by 20 s of deadtime. Images collected from the plate detector were radially integrated using GSAS-II and calibrated using a silicon standard.

Sequential Rietveld refinements were conducted on *in situ* SXPRD datasets using TOPAS Professional v6.[4] Lattice parameters, background terms, and scale factors were refined for each phase as a function of temperature, while atomic coordinates and occupancies were held constant at the initial values of the reference structure. A weighted scale factor (W.S.F.) Q was calculated for each phase p as a product of scale factor S, cell volume V, and cell mass M: $Q_p = S_p \bullet V_p \bullet W_p$.[5] We note that amorphous and liquid phases are inherently not observed in powder diffraction measurements and therefore cannot be accurately included in this analysis. A Lorentzian size broadening term was refined for each phase to model the peak shape using the pattern showing the greatest intensity of the relevant phase; this term was then fixed for the sequential refinements to better account for changes in intensity. To help stabilize the sequential refinement, isotropic displacement parameters (B_{iso}) were fixed at 1 Å^2 for all atoms, but we note that this is likely not physical for a variable temperature investigation.

Ex situ powder X-ray diffraction analysis of Zn_3WN_4

The products of all reactions were characterized by powder X-ray diffraction (PXRD). Laboratory X-ray diffraction patterns were collected on a Rigaku Ultima IV diffractometer with Cu Kα X-ray radiation at room temperature. All samples were initially prepared for PXRD measurements inside the glovebox; powder was placed on off-axis cut silicon single crystal wafers to reduce background scattering and then covered with polyimide tape to impede exposure to atmosphere. After Zn_3WN_4 was determined to be moderately air stable, PXRD patterns were collected without polyimide tape to decrease the background signal.

High resolution synchrotron powder X-ray diffraction (SPXRD) measurements were conducted at beamline 28-ID-2 of the National Synchrotron Light Source II (λ = 0.1821 Å) at Brookhaven National Laboratory. Samples were sealed under vacuum in quartz capillaries, which were then nested in Kapton capillaries. Data were collected for 60 seconds at T = 25 °C while spinning. Scattered photon intensity was measured using a Perkin-Elmer XRD 1621 Digital Imaging Detector. The data were reduced using Dioptas.[6] Pawley fits and subsequent Rietveld refinements were conducted using TOPAS Academic.[4]

Rietveld refinements were conducted for the laboratory PXRD and SPXRD patterns using TOPAS and TOPAS Academic, v6 (Bruker AXS).[4] Reference structures were sourced from the Inorganic Crystal Structure Database (ICSD). The $Pmn2_1$ Zn_3MoN_4 structure (ICSD Col. Code 255744) was used as a starting model for $Pmn2_1$ Zn_3WN_4, with the Mo replaced by W.[7] For the cation disordered $P6_3mc$ Zn_3WN_4 structure, $P6_3mc$ ZnO was used as a starting model, with atomic occupancies adjusted to match the stoichiometry of Zn_3WN_4, and lattice parameters adjusted to match the SPXRD pattern. For each structure, lattice parameters, isotropic displacement parameters, and general atomic coordinates were refined. For some models, Zn and W occupancy were refined as detailed in the Discussion section and the Supplemental (Figure S7). Structural visualizations and reference PXRD patterns were generated using VESTA.[8]

Compositional, thermodynamic, and property measurements

The composition of nominal Zn_3WN_4 was measured by X-ray Fluorescence spectroscopy (XRF) and combustion analysis. Cation composition was quantified by XRF using a Bruker M4 Tornado with a Rh X-ray source. Samples were pelletized and XRF spectra were collected at 4 points across the pellet. Zn and W ratios were quantified from each spectra using the Bruker M4 software.

Differential scanning calorimetry (DSC) experiments were conducted using a Q20 system from TA Instruments. Samples were prepared in an Ar-filled glovebox. Samples (ca. 10 mg) were loaded into aluminum pans, which were crimped closed with an aluminum lid. The reference pan was also crimped closed under argon. Pans were then transferred out of the glovebox for measurement, and data were collected upon ramping up to 400 °C at a rate of 10 °C/min. Thermodynamic calculations for reaction enthalpies (ΔH_{rxn}) were conducted using formation enthalpy (ΔH_f) values reported in the Materials Project database.[9,10] Chemical potential diagrams were generated in the method of Yokokawa[11] as detailed by Todd et al.[12] and implemented in pymatgen (v2023.9.10).[13]

UV-vis measurements were conducted on a Cary 6000 UV-Vis-NIR spectrometer. PTFE was used as a white reflectance standard. Absorbance was calculated with the Kubelka-Munk transformation, $k/s = (1 - R)^2 / 2R$ (where R is the reflectance, k is the apparent absorption coefficient, and s is the apparent scattering coefficient).

DC susceptibility data were measured on a Quantum Design Physical Properties Measurement System (PPMS) from $T = 2$ to 305 K in applied fields up to $\mu_0 H = 14$ T.

Additional PXRD measurements

Laboratory PXRD confirms the phase purity of the precursors that were synthesized for our metathesis reactions (Figure S1). All peaks index to the reference structures from the ICSD. The broad background at 2θ < 35° comes from the polyimide tape used to protect the powder from air and moisture. This background suppresses the measured peak intensities for peaks at 2θ < 35°, leading to the poor fit for that region of the pattern.

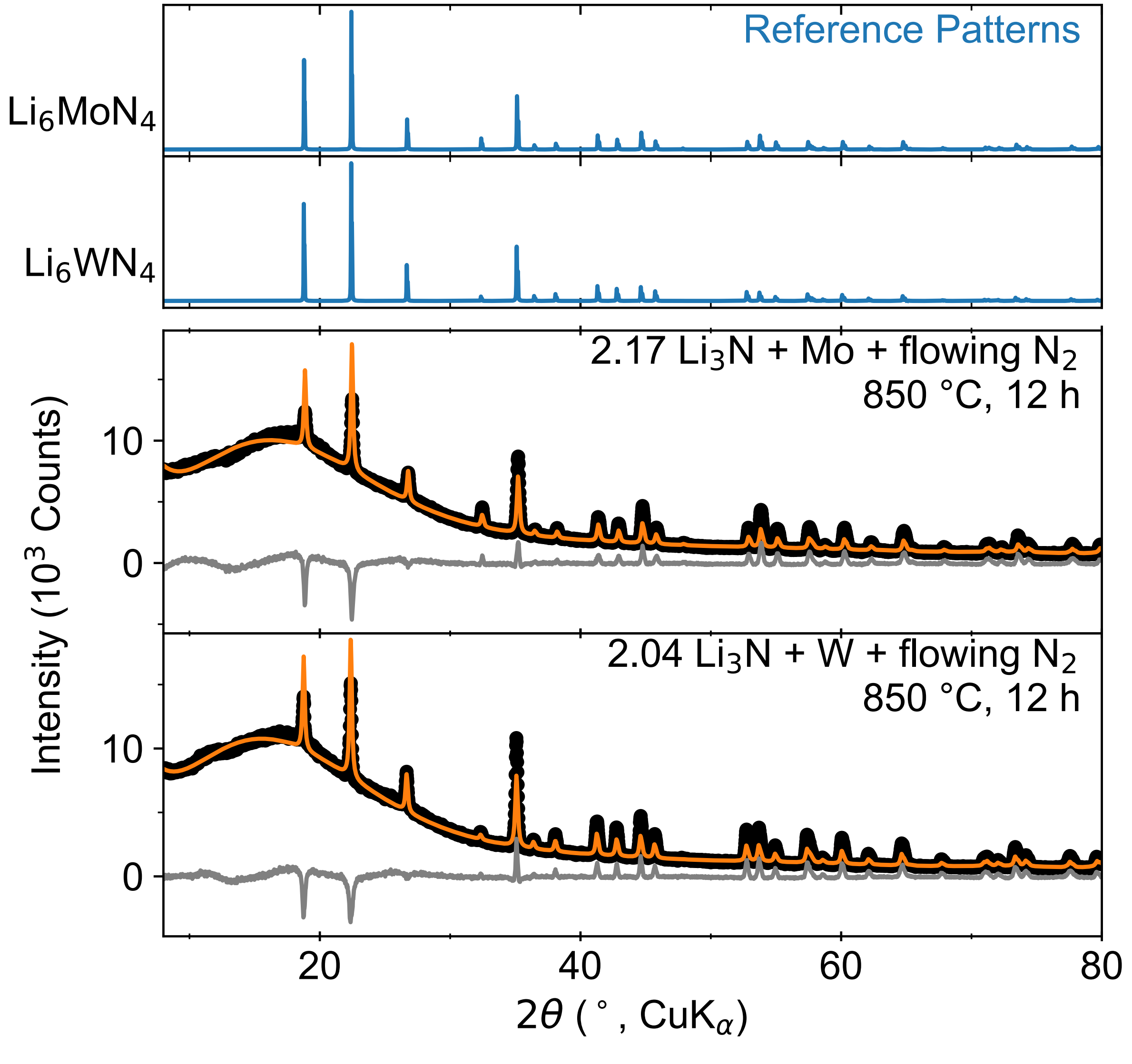

Figure S1. PXRD patterns and Rietveld refinement fits for the Li_6MoN_4 and Li_6WN_4 precursors. Simulated reference patterns are shown in the top boxes (ICSD Col. Code 66095 and 66096 for Li_6MoN_4 and Li_6WN_4, respectively).

In situ variable temperature SPXRD measurements

Figures S2 and S3 show the *in situ* variable temperature SPXRD heatmaps for the $ZnCl_2$ and ZnF_2 reactions, respectively. The analogous $ZnBr_2$ reaction is shown in the main text (Figure 1). These data were used for sequential Rietveld analysis, which is presented in Figure 4 in the main text.

In situ SPXRD of the 3 $ZnCl_2$ + Li_6WN_4 reaction (Figure S2) proceeds similarly to the $ZnBr_2$-based reaction shown in the main text (Figure 1). Bragg peaks arising from the precursors (3 $ZnCl_2$ + Li_6WN_4) stay steady up to 200 °C, where they begin to decrease in intensity. Simultaneously, Bragg peaks for Zn_3WN_4, LiCl, and Li_2ZnCl_4 begin to increase in intensity. The set of Bragg peaks corresponding to the Li_2ZnCl_4 phase fade out by 310 °C. We suspect that Li_2ZnCl_4 is not an essential intermediate but rather the product of a transient side reaction between the precursor $ZnCl_2$ and product LiCl. The main factor influencing reactivity is the liquidus line in the LiCl-$ZnCl_2$ system, with the lowest eutectic being at 275 °C.[14] The intensity of the Bragg peaks corresponding to the LiCl phase reaches a maximum near ca. 340 °C, and then slowly decreases in intensity up to 550 °C (LiCl melting point is 605 °C).[15] Zn_3WN_4's Bragg peaks remain approximately constant in intensity above 300 °C, persisting through the duration of the heating process. These processes are consistent with the following reactions:

$Li_6WN_4 + 3\ ZnCl_2 \rightarrow Zn_3WN_4 + 6\ LiCl$

$2\ LiCl + ZnCl_2 \rightarrow Li_2ZnCl_4$

Figure S3 shows that the 3 ZnF_2 + Li_6WN_4 reaction does not yield Zn_3WN_4 until 565 °C, a much higher temperature than the $ZnCl_2$ and $ZnBr_2$ reactions. Initial reactivity begins near 310 °C, indicated by a decrease in intensities for the set of Bragg peaks corresponding to Li_6WN_4 and ZnF_2. This initial reactivity is well below the melting point of ZnF_2 (872 °C).[15] Concurrent with this initial reaction, an unknown phase briefly grows in (between 310 °C and 404 °C). Extrapolating from the $ZnBr_2$ and $ZnCl_2$ reactions, the phase is likely a Li-Zn-F intermediate, but ternary fluorides in this space are poorly characterized. Li_2ZnF_4 has been reported, but the structure is not well described and the unit cell does not match the unknown phase. This intensity of the Bragg peaks arising from the intermediate phase decreases to zero by 404 °C, above which Li_6WN_4 is the only crystalline phase up to 565 °C. At this point, Li_6WN_4 fades out and Zn_3WN_4, LiF, and a rocksalt phase fit with WN grow in. The presence of this rocksalt phase indicates that the higher reaction temperature and greater exothermicity of the ZnF_2 reaction (compared with the Cl and Br versions) leads to a substantial degree of decomposition of the Zn_3WN_4 phase. Therefore, this reaction was not explored further.

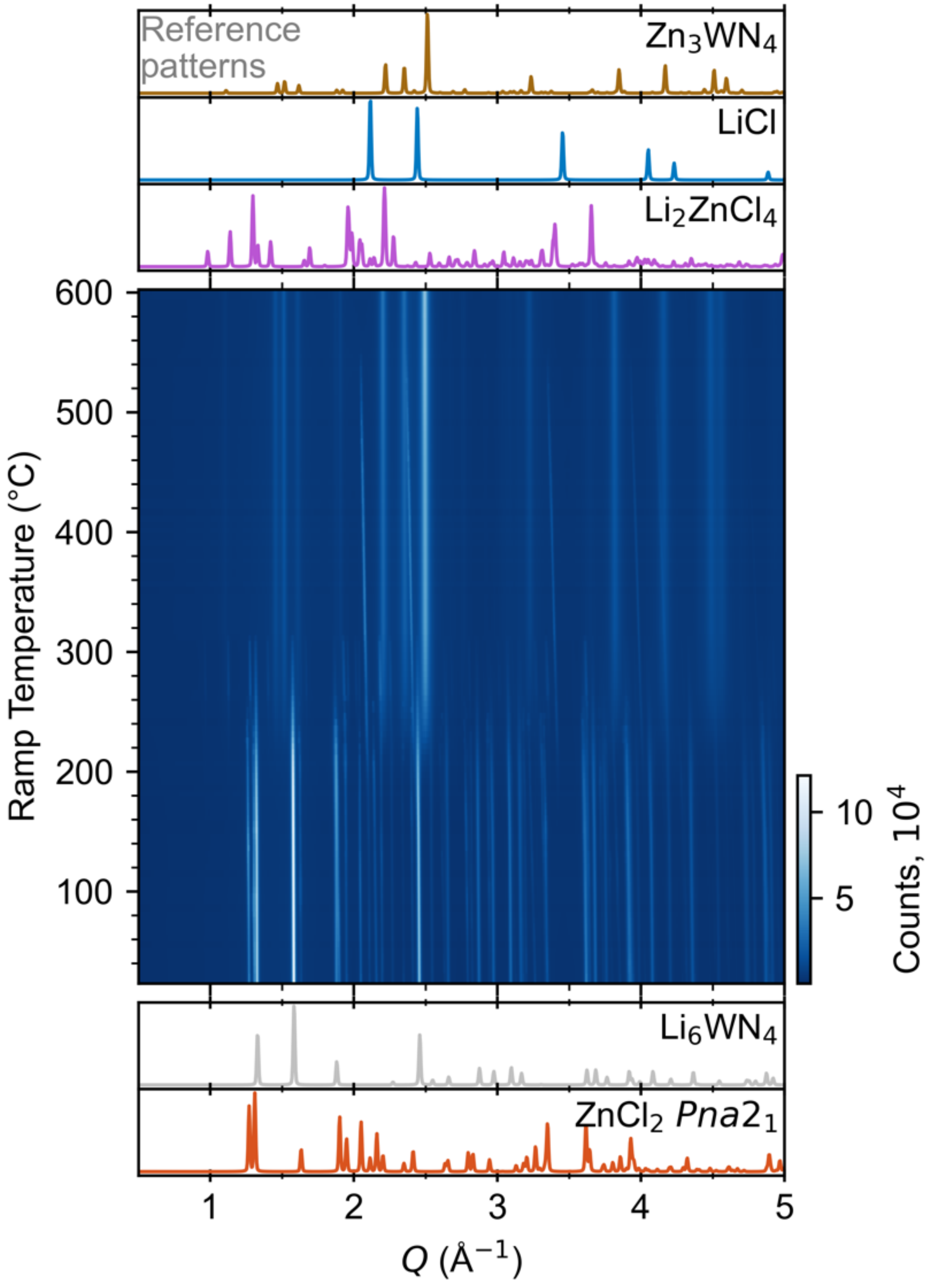

Figure S2. Heatmap of *in situ* SPXRD data upon heating 3 $ZnCl_2$ + Li_6WN_4 at +10 °C/min. Reference patterns for the reactants and products/intermediates are simulated at the bottom and top, respectively (ICSD Col. Codes 2459 for $ZnCl_2$, 66096 for Li_6WN_4, 402399 for Li_2ZnCl_4, and 27981 for LiCl).[1]

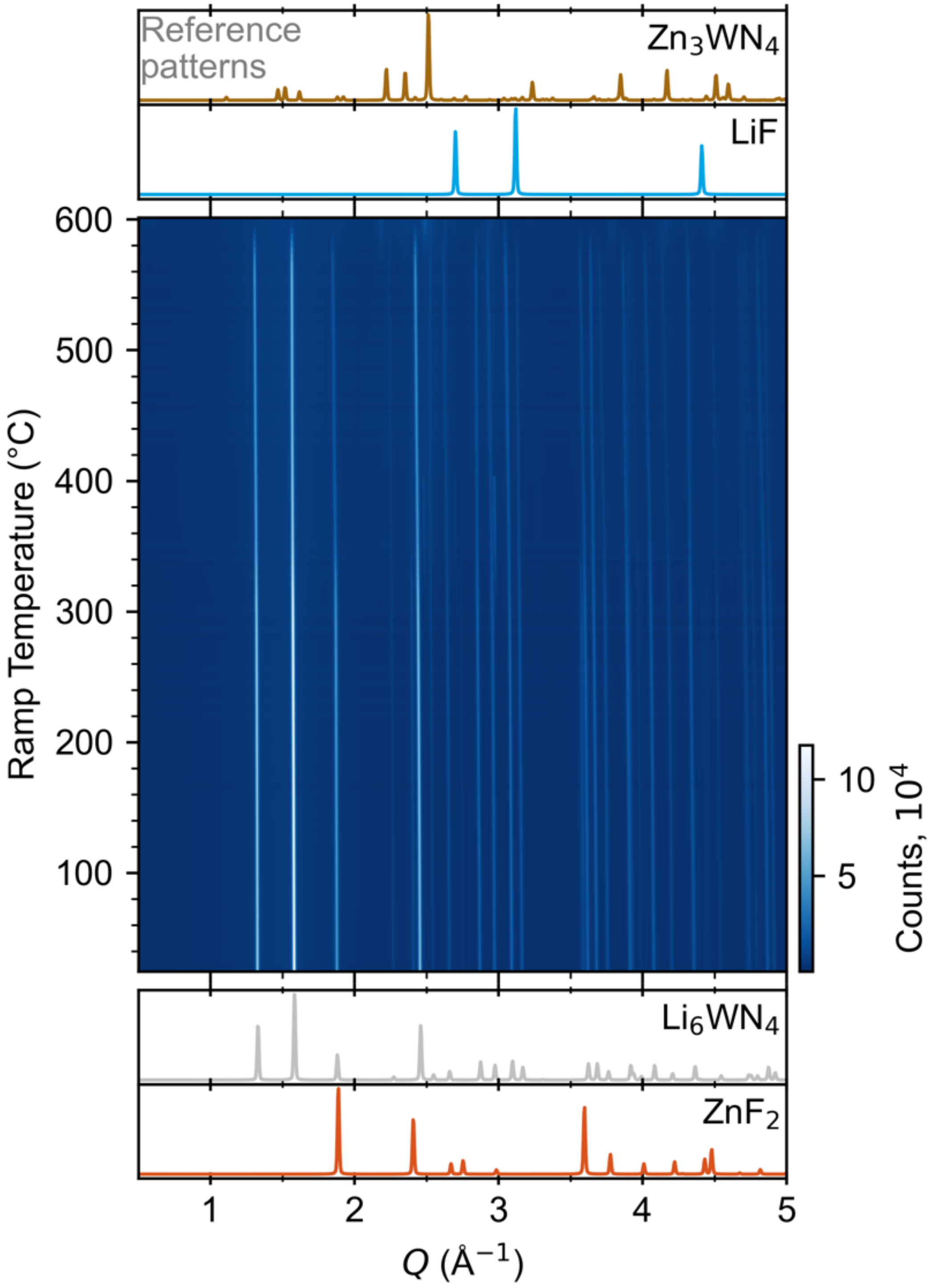

Figure S3. Heatmap of *in situ* SPXRD data upon heating 3 ZnF_2 + Li_6WN_4 at +10 °C/min. Reference patterns for the reactants and products/intermediates are simulated at the bottom and top, respectively. (ICSD Col. Codes 9169 for ZnF_2, 66096 for Li_6WN_4, 18012 for LiF).[1]

Synthesis of Zn_3MoN_4

The synthesis strategy used for Zn_3WN_4 was also applied to synthesize Zn_3MoN_4, but the product exhibited partial decomposition. Analysis of the SPXRD pattern collected for the Mo analog of Li_6WN_4, synthesized via the reaction $Li_6MoN_4 + 3\ ZnBr_2 \rightarrow Zn_3MoN_4 + 6\ LiBr$, suggests phase decomposition (Figure S4). In addition to the desired Zn_3MoN_4 (56 mol%), Rietveld analysis of High resolution SPXRD data show that a rocksalt (RS) structure fit as $ZnMoN_{0.5}$ also forms (23 mol%), along with a Zn impurity (20 mol%). This RS phase exhibits a substantially larger lattice parameter (a = 4.7106(3) Å) than the defect-RS phase Mo_2N (a = 4.16 Å to 4.19 Å).[17,18] Therefore, we hypothesize it may be a $(Zn,Mo)N_x$ material, as octahedra Zn^{2+} has a substantially larger ionic radius (0.74 Å) than octahedral Mo^{x+} (0.65 Å for Mo^{4+}, 0.69 Å for Mo^{3+}).[19] The rocksalt $ZnMoN_{0.5}$ phase was created from a $Fm\bar{3}m$ Mo_2N starting model. Rietveld analysis with the composition of $ZnMoN_{0.5}$ provides a reasonable fit. Further analysis of this material is beyond the scope of this manuscript. Additional minor peaks that we have not indexed are present (possibly higher order oxides). These impurity phases suggest that Zn_3MoN_4 is less stable at elevated temperatures than Zn_3WN_4. This decomposition occurs despite the excess $ZnBr_2$ which was intended to serve as a heat sink during the exothermic reaction. Despite the partial decomposition of the phase, Zn_3MoN_4 is still the major phase in the pattern. As with Zn_3WN_4, the SPXRD pattern for Zn_3MoN_4 shows evidence of cation-ordering in the form of the $Pmn2_1$ reflections at low angle: e.g., (010), (110), (101), (011). However, these reflections are weaker than in the W case, owing to the lower scattering factor of Mo compared to W. We focused our work on Zn_3WN_4 because W scatters X-rays more strongly than Mo (facilitating characterization) and because our Zn_3WN_4 products exhibited higher phase purity.

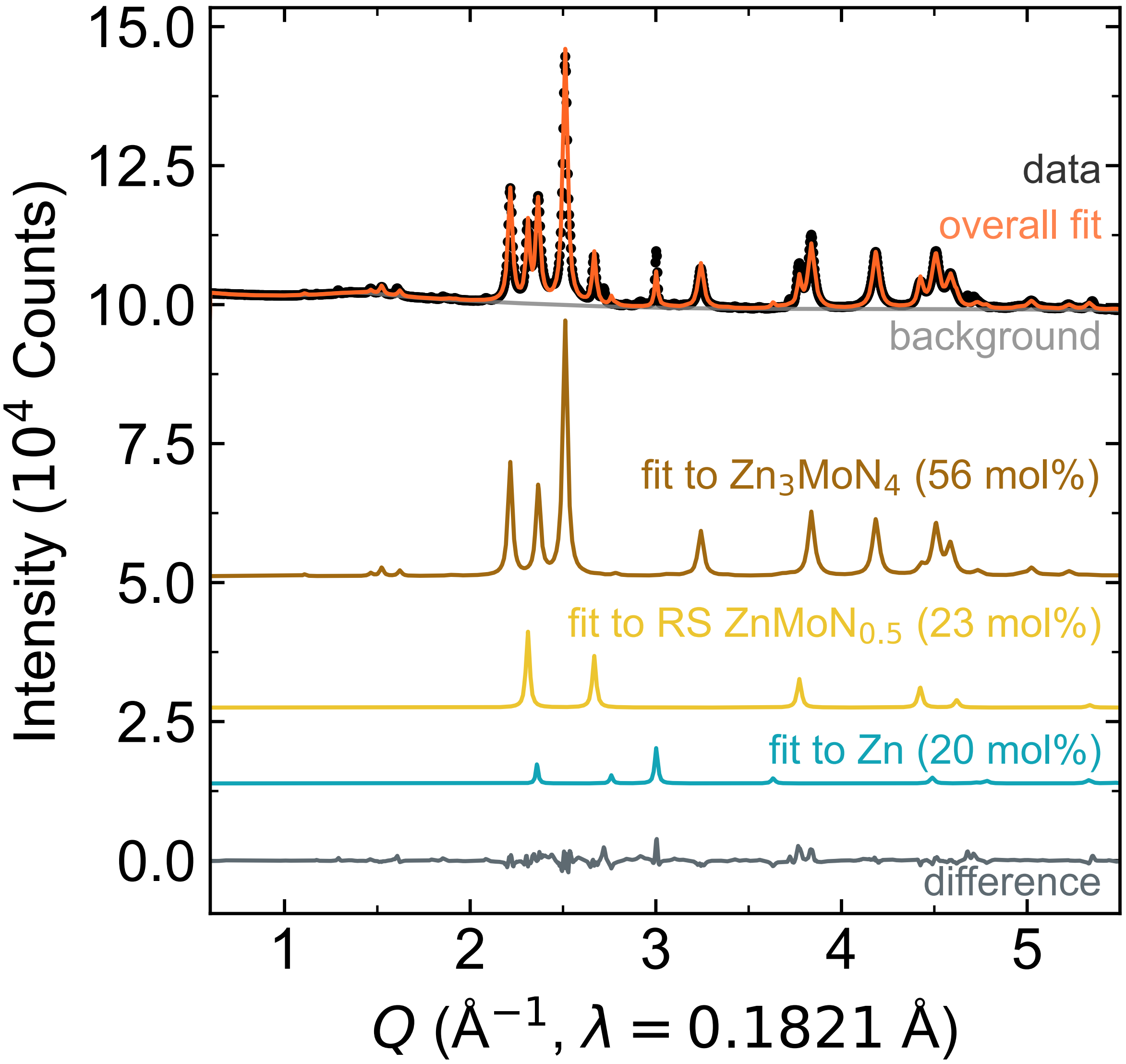

Figure S4. SPXRD pattern and Rietveld refinement of the washed products from the reaction between Li_6MoN_4 + 4.2 $ZnBr_2$ (excess $ZnBr_2$).

DSC measurements

The rapid, exothermic nature of the $ZnCl_2$-based reaction produced an odd curvature (peak b-ii) in the temperature vs. heat flow trace shown in Figure 3b. The reaction releases so much heat in such a short time that the aluminum DSC pan and stage increase in temperature by approximately 16 °C before cooling back down to the programed temperature (Figure S5b). This peak in the time vs. temperature trace appears as the curved peak (peak b-ii) in the temperature vs. heat flow trace (Figure 3b). In contrast, the $ZnBr_2$ reaction does not noticeably affect the temperature of the DSC system (Figure S5a).

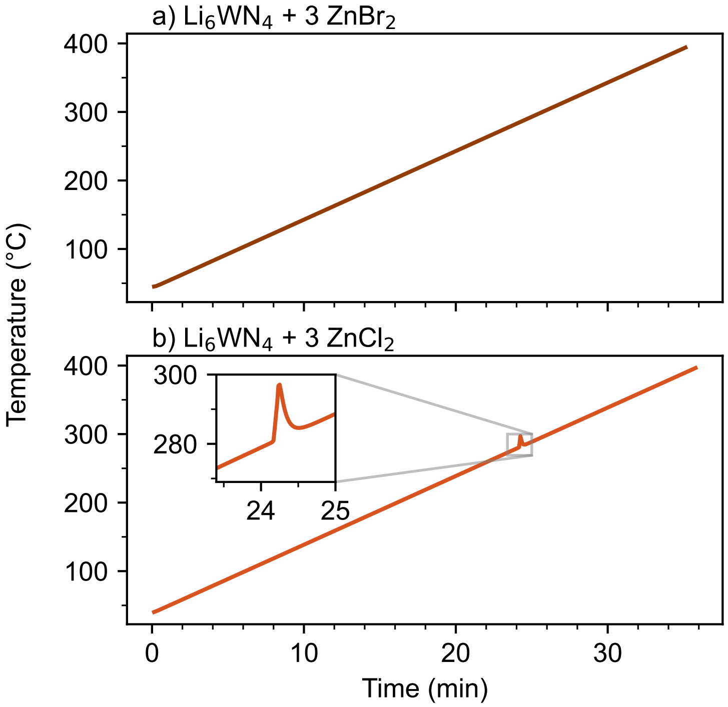

Figure S5. The time vs. temperature trace from the DSC experiments on reaction mixtures of a) Li_6WN_4 + $ZnBr_2$ and b) Li_6WN_4 + $ZnCl_2$. Corresponding temperature vs. heat flow traces are shown in the main text (Figure 3).

Evidence of decomposition in the 3 $ZnCl_2$ + Li_6WN_4 reaction

Figure S6 shows that a trace Zn impurity can be detected in reactions between Li_6WN_4 + 3.1 $ZnCl_2$, even when heated at low temperatures (ca. 250 °C). This impurity makes the powder appear grey in color. The Zn is likely produced via decomposition of Zn_3WN_4 during the highly exothermic reaction (See DSC measurements, Figure 3). Surprisingly, we do not observe W or WN. This may indicate that tungsten remains in the Zn_3WN_4 phase (which would then be Zn-poor), or that the W (or WN) is amorphous.

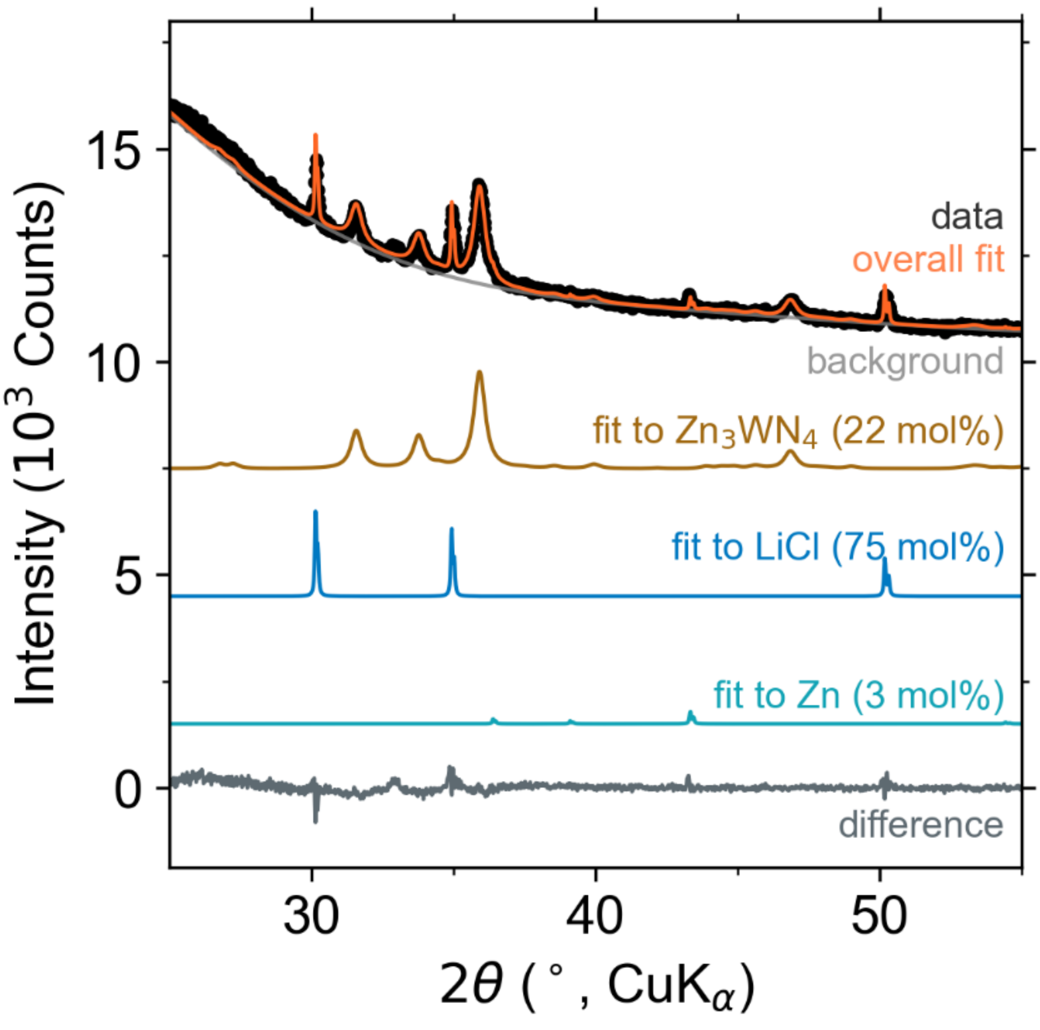

Figure S6. Laboratory PXRD measurement of the product of Li_6WN_4 + 3.1 $ZnCl_2$ heated at +5 °C/min to 250 °C for a 10 h dwell.

Chemical potential diagrams

Chemical potential diagrams show the relative stability of Zn_3WN_4 in the Li-Zn-W-N phase space. The Gibbs free energy is calculated as a function of temperature,[20] revealing that Zn_3WN_4 is calculated to be thermodynamically stable (i.e., on the convex hull) at the synthesis temperature of 300 °C (Figure S7a,b), but becomes destabilized relative to competing phases (W, W_2N_3, N_2, Zn) by 700 °C (Figure S7c,d). Therefore, Zn_3WN_4 would likely decompose near 700 °C at 1 atm of N_2. To represent a 4-dimensional space in 3-dimensions, we project down the Li-W-N space onto its intersection with the Zn-W-N axes, so the stability region of Li_6WN_4 is shown as a grey volume. The Li_6WN_4 chemical potential space intersects that of Zn_3WN_4 (Figure S6a,b), indicating that these two phases are thermodynamically stable against one another.[11] Prior work on oxides[12] and nitrides[21] has shown that this intersection in chemical potential space means interdiffusion can effectively occur between the two phases, allowing for the formation of the targeted phase (Zn_3WN_4 in this case) without nucleating an intermediate at the interface.

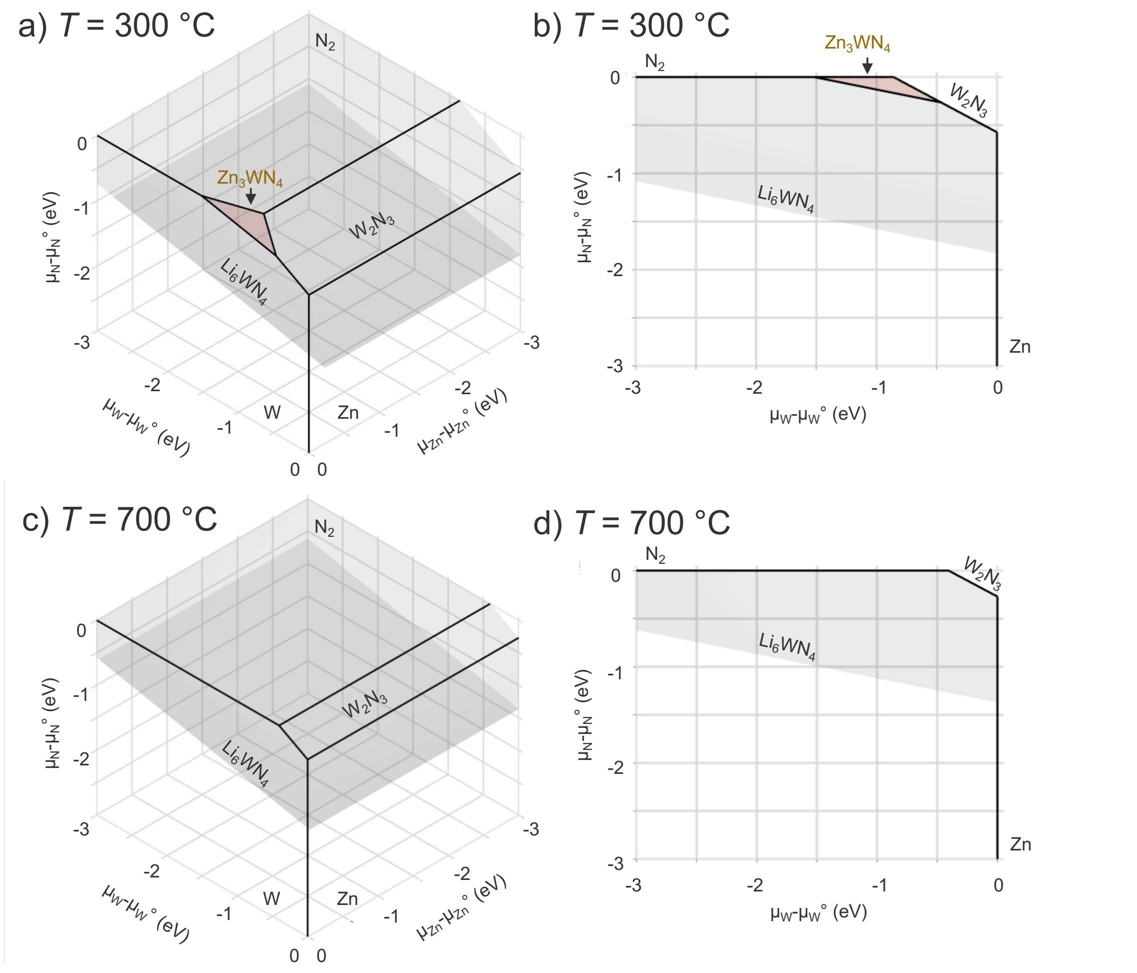

Figure S7. Calculated chemical potential diagrams for the (Li)-Zn-W-N system at a,b) 300 °C and c,d) 700 °C. Panels a and c show a 3D perspective while panels b and d show the space on the W-N chemical potential axes.

Compositional characterization

X-ray Fluorescence (XRF) spectroscopy identified a Zn:W ratio of 3.8:1, in excess of the expected 3:1 ratio for Zn_3WN_4. XRF was conducted on pelletized powder after washing away the bromide byproduct. The excess zinc may be incorporated in the wurtzite-derived lattice, as suggested by Rietveld analysis (discussed below). A representative raw spectrum is shown in Figure S8. Spectra were collected at 4 different points across the pellet, and each spectrum was fit using the Bruker software to quantify Zn and W atomic ratios. The Zn:W ratio of 3.8:1 was calculated by averaging the Zn:W values from the 4 spectra.

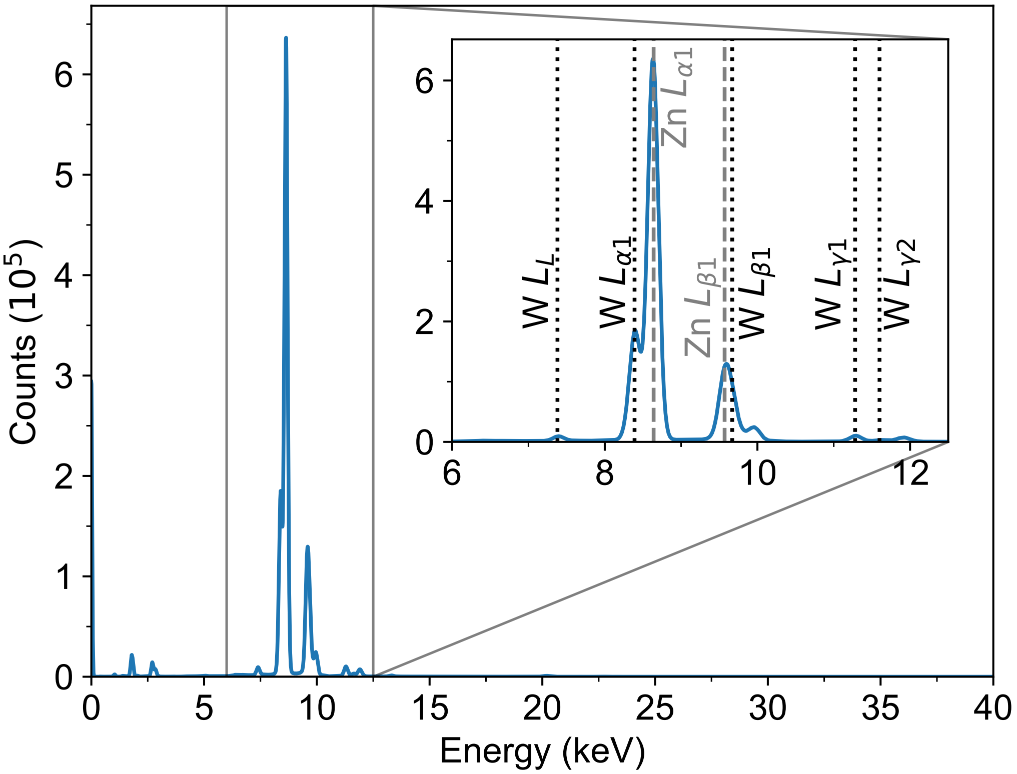

Figure S8. Representative XRF spectrum from the surface of a Zn_3WN_4 pellet. The inset shows the characteristic X-ray lines used in the fitting software.

Structural models for the high resolution SPXRD measurements

The refined lattice parameters for Zn_3WN_4 are shown in Tables S1.

Table S1. Refined atomic coordinates for Zn_3WN_4 in space group $Pmn2_1$ from the SPXRD shown in Figure 5a. The unit cell parameters refined to a = 6.5602(8) Å, b = 5.6813(7) Å, and c = 5.3235(2) Å. R_{exp}= 1.182 %, R_{wp} = 3.989 %, R_p = 2.802 %, GOF = 3.374. This structure is visualized in Figure 5d.

Site	Wyckoff	Atom	*x*	*y*	*z*	Occupancy	B_{iso} (Å^2)
Zn1	4b	Zn	0.251(1)	0.834(1)	0.697(1)	1.00(1)	0.10(4)
Zn1	4b	W	0.251(1)	0.834(1)	0.697(1)	0.00(1)	0.10(4)
Zn2	2a	Zn	0	0.337(6)	0.698(1)	1.00(1)	0.20(7)
Zn2	2a	W	0	0.337(6)	0.698(1)	0.00(1)	0.20(7)
W1	2a	W	0	0.670(2)	0.221(1)	0.83(2)	0.44(4)
W1	2a	Zn	0	0.670(2)	0.221(1)	0.17(2)	0.44(4)
N1	4b	N	0.765(2)	0.846(3)	0.095(1)	1	0.30(18)
N2	2a	N	0	0.330(4)	0.116(2)	1	0.4(3)
N3	2a	N	0	0.678(4)	0.555(2)	1	0.4(3)

We considered several structural models of Zn_3WN_4 when conducting Rietveld analysis against our high resolution SPXRD patterns (Table S2). Several terms were allowed to vary for each approach: sample displacement, lattice parameters, size broadening (Lorentzian), strain broadening (Lorentzian), isotropic displacement parameters, and a 15-term background Chebyshev polynomial. Our most robust model was a single-phase model that allowed for a small degree of cation disorder for $Pmn2_1$ Zn_3WN_4, but with each cation site fixed to full occupancy (e.g., the Zn1 site was refined with Zn occupancy set to 1-x and W occupancy x). This model resulted in an R_{wp} of 3.989 % and is shown in Figure 5, Table S1, and Figure S9a. For comparison, a simpler model of $Pmn2_1$ Zn_3WN_4 with fixed cation occupancies (e.g., the Zn1 site fixed with 1.0 Zn occupancy) gave a significantly worse fit to the data (R_{wp} = 4.638 %, Figure S9b). Atomic positions were allowed to refine for both these single phase models. However, our diffuse reflectance spectroscopy measurements suggest that the material is not a perfectly cation-ordered phase. While the small of cation disorder in the $Pmn2_1$ Zn_3WN_4 structure can explain the optical data,[22] we also considered a two-phase model.

Given the two distinct absorption onsets shown in the diffuse reflectance spectrum (Figure 5), we also considered a two-phase model in our Rietveld refinements. For the first two-phase model, we started with the $Pmn2_1$ Zn_3WN_4 from the fixed cation occupancy models. We then fixed atomic positions. Next, we created a model for cation disordered Zn_3WN_4 in a $P6_3mc$ structure (i.e., the wurtzite structure type), and fixed the lattice parameters of the disordered phase (dis) as functions of the refined lattice parameters for the ordered phase (ord): $a_{dis} = 0.5a_{ord}$ and $c_{dis} = c_{ord}$. This relationship holds true for the order-disorder transition of the related wurtzite-derived material, $ZnGeN_2$.[23] This disordered structure is consistent with the cation-disordered Zn_3WN_4 synthesized via thin film sputtering.[24] We then refined the size and strain broadening for both phases. The disordered phase exhibited broader peaks (meaning smaller size, larger strain) than the ordered phase, but this analysis is qualitative rather than quantitative as the broadening contribution from the beam profile could not be reliably refined. This refinement resulted in 78 mol% phase fraction

of $Pmn2_1$, 22 mol% for $P6_3mc$, and an R_{wp} value of 3.953 % (Figure S9a), comparable to the single-phase model. However, we chose not to present this model in the main text, given that the extra complexity does not substantially improve the goodness of fit.

The best fit was obtained via a two-phase model, but with non-physical lattice parameters. Allowing the $P6_3mc$ lattice parameters to freely refine results in the best fit we obtained by the Rietveld method (R_{wp} = 3.742 %). However, the model is likely non-physical. In this model, the refined *c* lattice parameter for this $P6_3mc$ phase increases substantially (5.451(2) Å) compared to the $Pmn2_1$ phase (c = 5.3228(2) Å), which is not consistent with prior studies of order-disorder transitions in wurtzite-derived structures (e.g., $ZnGeN_2$).[23] The *c* lattice parameters of $ZnGeN_2$ are identical in the $Pna2_1$ (the ordered structure) and $P6_3mc$ (the disordered structure), because cation disorder does not affect the layer spacing of the hcp anions along the (00*l*) direction. This analysis reveals ambiguities in these two-phase models.

Given the limitations of the two-phase models, we posit that the single phase model provides the most reliable fit to the SPXRD data without over-fitting the pattern.[25] Yet, the SPXRD measurements probe the long range average ordered structure. Local ordering—which we do not probe here—may influence the optical absorption properties shown in Figure7. The impact of local ordering on optical properties has been characterized in the halide perovskite $CsSnBr_3$,[26] in Fe doped $SrTiO_3$,[27] and in carbon coated FeF_3.[28]

Table S2. Summary of models considered for the high resolution SPXRD data for Zn_3WN_4.

**Rietveld approach**	**Space group**	**Composition**	**R_{wp} (%)**	**Figure**
Single phase.<br>Refined cation occ.	$Pmn2_1$	$Zn_{3.17}W_{0.83}N_4$	3.989	1a and S6a
Single phase.<br>Fixed cation occ.	$Pmn2_1$	Zn_3WN_4	4.638	S6b
Two phases.<br>Fixed cation occ.<br>Fixed *a* and *c* for $P6_3mc$	$Pmn2_1$<br>$P6_3mc$	Zn_3WN_4<br>$Zn_{0.75}W_{0.25}N$	3.953	S7a
Two phases.<br>Fixed cation occ.<br>Refined *a* and *c* for $P6_3mc$	$Pmn2_1$<br>$P6_3mc$	Zn_3WN_4<br>$Zn_{0.75}W_{0.25}N$	3.742	S7b

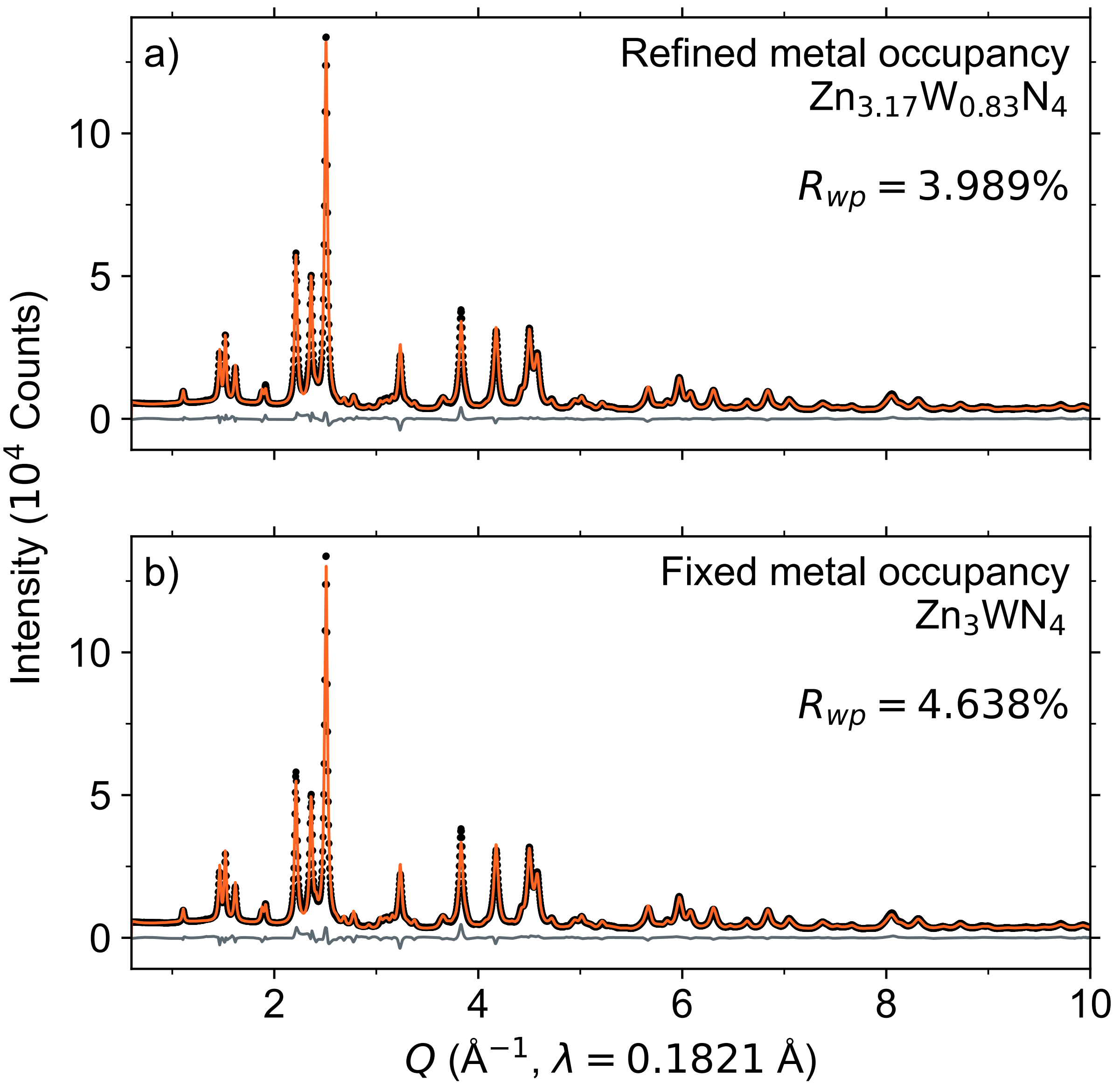

Figure S9. The high resolution SPXRD measurement of the Zn_3WN_4 sample fit with single-component models of *Pmn*2_1 with a) the occupancies of the metal sites refined to $Zn_{3.17}W_{0.83}N_4$ (also shown in Figure 4), and b) fixed metal site occupancy at cation-ordered Zn_3WN_4.

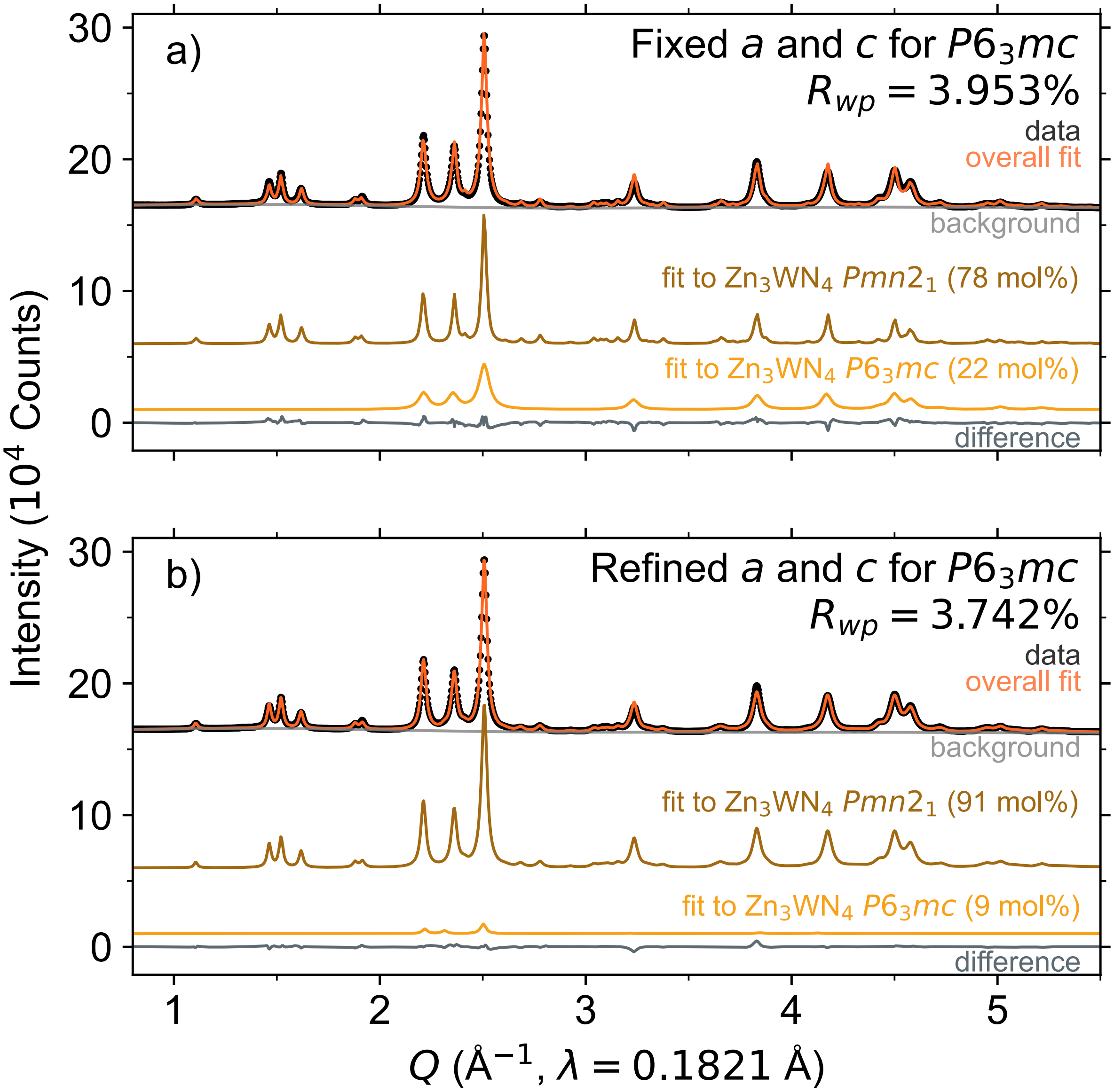

Figure S10. Rietveld refinement of the high-resolution SPXRD data of Zn_3WN_4 using a two-component model with a) the $P6_3mc$ lattice parameters fixed relative to the $Pmn2_1$ values and b) the $P6_3mc$ lattice parameters freely refined.

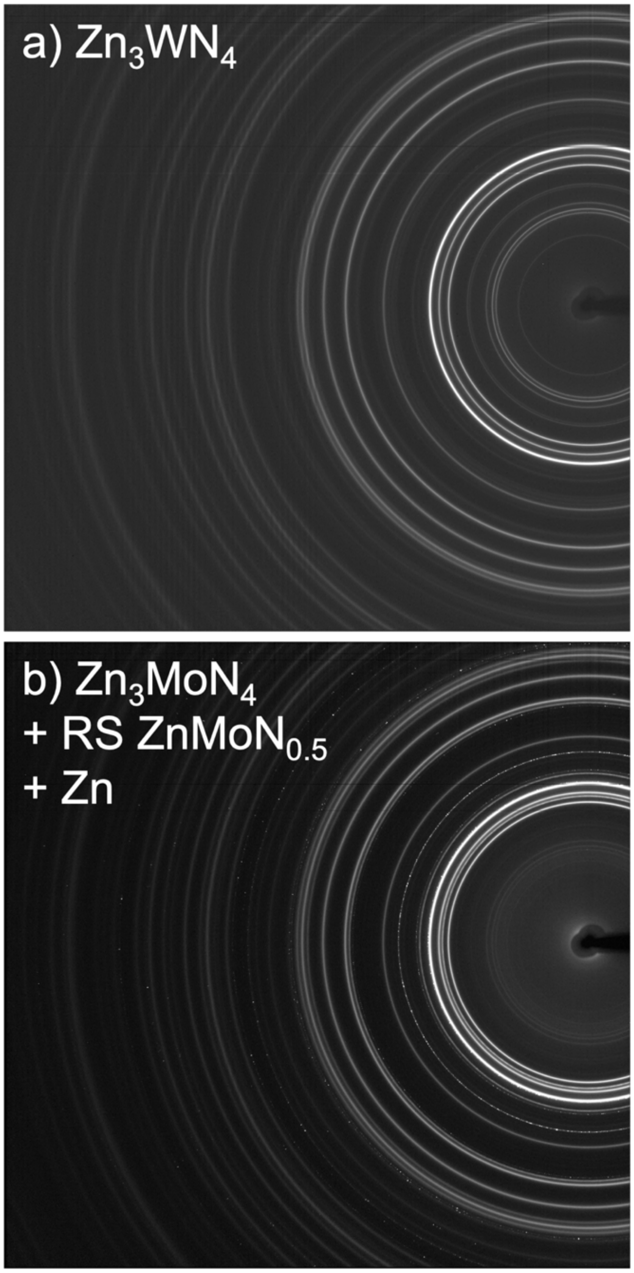

Figure S11. High resolution SXPRD 2D diffraction images from the 2D detector for the integrated patterns shown in Figure 1 (a) Zn_3WN_4 and (b) Zn_3MoN_4 + decomposition products rocksalt (RS) $ZnMoN_{0.5}$ and metallic Zn.

Qualitative inspection of the 2D diffraction images show that the diffraction rings for the Zn_3WN_4 powder are homogeneous (Figure S8). If rings with two different morphologies were present, this would suggest the presence of two distinct phases with different crystallinity, size, and strain. That we see only one morphology of ring in the 2D detector image supports either a single phase or multiple phases with nearly identical crystallinity, size, and strain. In our Rietveld refinement that modeled the data using two phases, the fit to the data is significantly worse when we constrain the size and strain broadening terms to be the same for both phases. These findings support our use of the single-phase model.

Magnetic susceptibility measurements

Magnetic measurements were performed on Zn_3WN_4 using a Quantum Design Physical Property Measurement System (PPMS). A powder sample of Zn_3WN_4 was loaded into a small packet (0.001125 mg) and secured inside a plastic straw for the measurement. Magnetic susceptibility (χ) of Zn_3WN_4 was measured as a function of temperature shows largely diamagnetic behavior with a trace paramagnetic impurity (Figure S12a). Similarly, magnetization (M) as a function of applied field (H) at 2 K shows that diamagnetism dominates the field-dependent magnetization (Figure S12b). These findings are inconsistent with pure Zn_3WN_4, which should be purely diamagnetic. Zn impurities, if present, would also give a diamagnetic response. The paramagnetic component suggests the possibility of a reduced tungsten species (e.g., W^{5+}), possibly as a sub-nitride (e.g., $Zn_3WN_{4-\delta}$), an oxynitride impurity (e.g., $Zn_3WN_{4-x}O_x$), or a W-rich phase (e.g., $Zn_{3-\delta}W_{1+\delta}N_4$).

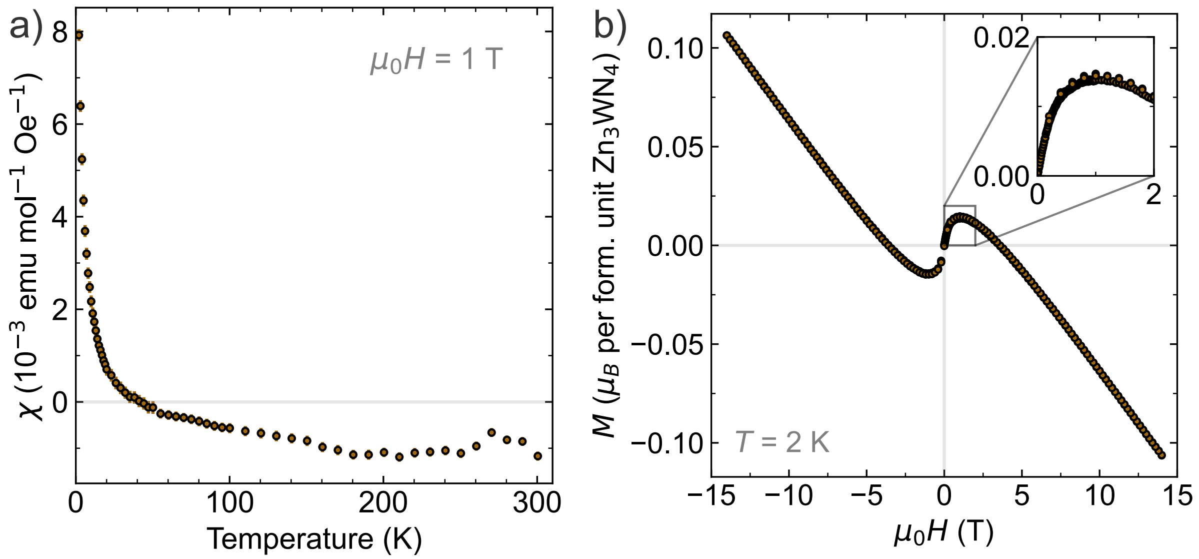

Figure S12. A) Magnetic susceptibility (χ) of Zn_3WN_4 as a function of temperature with an applied field (μ_0H) of 1 T. b) Magnetization (M) as a function of μ_0H at 2 K. The grey outline in (b) is shown in more detail via the inset.

Full structure visualizations of Li_6WN_4 and Zn_3WN_4

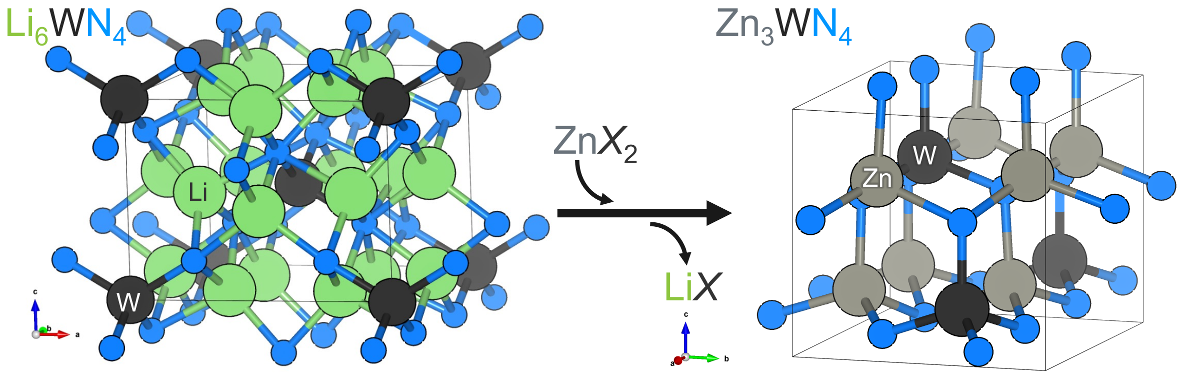

Figure S13. Reaction scheme showing the structure of Li_6WN_4 and Zn_3WN_4, with Li and Zn atoms included to complement the main text visualization (Figure 7).

References

[1] W. X. Yuan, J. W. Hu, Y. T. Song, W. J. Wang, Y. P. Xu, *Powder Diffr.* **2005**, *20*, 18–21.
[2] D. B. G. Williams, M. Lawton, *J. Org. Chem.* **2010**, *75*, 8351–8354.
[3] P. J. Chupas, K. W. Chapman, C. Kurtz, J. C. Hanson, P. L. Lee, C. P. Grey, *J. Appl. Crystallogr.* **2008**, *41*, 822–824.
[4] A. A. Coelho, *J. Appl. Crystallogr.* **2018**, *51*, 210–218.
[5] P. K. Todd, A. M. M. Smith, J. R. Neilson, *Inorg. Chem.* **2019**, *58*, 15166–15174.
[6] C. Prescher, V. B. Prakapenka, *High Press. Res.* **2015**, *35*, 223–230.
[7] E. Arca, S. Lany, J. D. Perkins, C. Bartel, J. Mangum, W. Sun, A. Holder, G. Ceder, B. Gorman, G. Teeter, W. Tumas, A. Zakutayev, *J. Am. Chem. Soc.* **2018**, *140*, 4293–4301.
[8] K. Momma, F. Izumi, *J. Appl. Crystallogr.* **2011**, *44*, 1272–1276.
[9] A. Jain, G. Hautier, S. P. Ong, C. J. Moore, C. C. Fischer, K. A. Persson, G. Ceder, *Phys. Rev. B* **2011**, *84*, 045115.
[10] A. Jain, S. P. Ong, G. Hautier, W. Chen, W. D. Richards, S. Dacek, S. Cholia, D. Gunter, D. Skinner, G. Ceder, K. A. Persson, *APL Mater.* **2013**, *1*, 011002.
[11] H. Yokokawa, *J. Phase Equilibria* **1999**, *20*, 258–287.
[12] P. K. Todd, M. J. McDermott, C. L. Rom, A. A. Corrao, J. J. Denney, S. S. Dwaraknath, P. G. Khalifah, K. A. Persson, J. R. Neilson, *J. Am. Chem. Soc.* **2021**, *143*, 15185–15194.
[13] S. P. Ong, W. D. Richards, A. Jain, G. Hautier, M. Kocher, S. Cholia, D. Gunter, V. L. Chevrier, K. A. Persson, G. Ceder, *Comput. Mater. Sci.* **2013**, *68*, 314–319.
[14] S. J. Shaw, G. S. Perry, *Thermochim. Acta* **1989**, *155*, 87–96.
[15] J. R. Rumble (ed.), *"Physical Constants of Inorganic Compounds" in CRC Handbook of Chemistry and Physics*, CRC Press/Taylor & Francis, Boca Raton, FL., **2023**
[16] C. Martin, A. Durif-Varambon, J.-C. Joubert, *Bull. Minéralogie* **1965**, *88*, 141–141.
[17] C. L. Bull, T. Kawashima, P. F. McMillan, D. Machon, O. Shebanova, D. Daisenberger, E. Soignard, E. Takayama-Muromachi, L. C. Chapon, *J. Solid State Chem.* **2006**, *179*, 1762–1767.
[18] B. Cao, J. C. Neuefeind, R. R. Adzic, P. G. Khalifah, *Inorg. Chem.* **2015**, *54*, 2128–2136.
[19] R. D. Shannon, *Acta Crystallogr. Sect. A* **1976**, *32*, 751–767.

[20] C. J. Bartel, S. L. Millican, A. M. Deml, J. R. Rumptz, W. Tumas, A. W. Weimer, S. Lany, V. Stevanović, C. B. Musgrave, A. M. Holder, *Nat. Commun.* **2018**, *9*, 4168.
[21] C. L. Rom, A. Novick, M. J. McDermott, A. A. Yakovenko, J. R. Gallawa, G. T. Tran, D. C. Asebiah, E. N. Storck, B. C. McBride, R. C. Miller, A. L. Prieto, K. A. Persson, E. Toberer, V. Stevanović, A. Zakutayev, J. R. Neilson, *J. Am. Chem. Soc.* **2024**, *146*, 4001–4012.
[22] J. J. Cordell, G. J. Tucker, A. Tamboli, S. Lany, *APL Mater.* **2022**, *10*, 011112.
[23] E. W. Blanton, K. He, J. Shan, K. Kash, *J. Cryst. Growth* **2017**, *461*, 38–45.
[24] W. Sun, C. J. Bartel, E. Arca, S. R. Bauers, B. Matthews, B. Orvañanos, B.-R. Chen, M. F. Toney, L. T. Schelhas, W. Tumas, J. Tate, A. Zakutayev, S. Lany, A. M. Holder, G. Ceder, *Nat. Mater.* **2019**, *18*, 732–739.
[25] B. H. Toby, *Powder Diffr.* **2006**, *21*, 67–70.
[26] D. H. Fabini, G. Laurita, J. S. Bechtel, C. C. Stoumpos, H. A. Evans, A. G. Kontos, Y. S. Raptis, P. Falaras, A. Van der Ven, M. G. Kanatzidis, R. Seshadri, *J. Am. Chem. Soc.* **2016**, *138*, 11820–11832.
[27] H. A. J. L. Mourão, O. F. Lopes, W. Avansi, M. J. M. Pires, S. Souza, C. Ribeiro, V. R. Mastelaro, *Mater. Sci. Semicond. Process.* **2017**, *68*, 140–146.
[28] N. Yamakawa, M. Jiang, B. Key, C. P. Grey, *J. Am. Chem. Soc.* **2009**, *131*, 10525–10536.
